# Multi-Morphogen Fields and Homeostatic Resource Allocation for Constrained Multiobjective Evolutionary Optimization

**DOI:** 10.3390/biomimetics11100719

**Published:** 2026-10-09

**Authors:** Xiaoguo Chen, Yongchao Li, Xingsen Li, Yue Yang

**Affiliations:** 1 School of Information Engineering, Sanming University, Sanming 365004, China; 20180137@fjsmu.edu.cn; 2 School of Information and Electrical Engineering, Heilongjiang Bayi Agricultural University, Daqing 163319, China; 3 Institute of Extenics and Innovation Methods, Guangdong University of Technology, Guangzhou 510006, China; lixs@gdut.edu.cn; 4 Key Laboratory of Engineering Material & Structure Reinforcement in Fujian Universities, Sanming University, Sanming 365004, China; 20180136@fjsmu.edu.cn

**Keywords:** constrained multiobjective optimization, multi-morphogen developmental fields, constraint relaxation, resource allocation

## Abstract

Effective constrained multiobjective optimization requires a search process to coordinate feasibility attainment, objective-space progress, and population distribution under a limited evaluation budget. This coordination becomes more difficult in dual-population frameworks when auxiliary search loses objective-direction information, applies unsuitable constraint tolerance, or consumes resources that no longer match its current contribution. Motivated by these limitations, we develop the Multi-Morphogen Field-Guided Constrained Multiobjective Evolutionary Algorithm (MFGCMOEA). Its main population is responsible for feasibility-oriented convergence, whereas the auxiliary population encodes each objective through a separate morphogen channel. Signals are propagated according to relative geometry in objective-rank space, allowing local search information to modify the developmental state of each candidate. These channel-wise states are then used to construct individual constraint buffers and multidimensional phenotypes, so promising infeasible directions can remain competitive without collapsing multiple objectives into a single scalar evaluation. Diversity regulation is applied only when truncation is required on the critical developmental front. For computational coordination, MFGCMOEA derives resource evidence from offspring developmental quality and realized auxiliary recruitment and combines it with a symmetric basal-growth term to prevent overly polarized offspring allocation. The method was evaluated on 47 benchmark problems from four suites using 30 independent runs. Among nine algorithms, MFGCMOEA obtained the best overall mean ranks, approximately 1.66 for IGD and 1.74 for HV. On the 26 problems with complete valid results for all methods, the Friedman test detected significant overall differences, while Holm-adjusted pairwise comparisons favored MFGCMOEA against all eight competitors with large rank-biserial effect sizes. Additional convergence, distribution, ablation, and diagnostic results further confirm the roles of the developmental-field representation and homeostatic resource regulation.

## 1. Introduction

The design and operation of engineering systems usually require multiple competing performance criteria to be coordinated under constraints imposed by physical laws, safety requirements, manufacturing conditions, or operating limits. For example, vehicle routing involves tradeoffs among transportation cost, time, and service quality [1], whereas knapsack problems require profit to be balanced against capacity limits [2]. Engineering designs involving robot grippers, trusses, and welded beams jointly consider structural performance, material consumption, and geometric or strength constraints [3,4,5]. Similar conflicts also arise in process system design and power electronics control [6,7]. A common feature of these problems is that no single globally optimal solution exists across the competing objectives. The feasible regions can be narrow, disconnected, or separated by large infeasible regions. A solution method must therefore approximate the true Pareto front, exploit search information outside the feasible region, and maintain convergence and solution distribution under a limited function evaluation budget. Constrained multiobjective optimization problems (CMOPs) have consequently become an important research topic in evolutionary computation [8].

Consider a constrained multiobjective minimization problem involving *m* objectives and *n* inequality constraints:

(1) minimizeF(x)=f1(x),…,fm(x)Ts.t.gj(x)≤0,j∈{1,2,…,n}s.t.x∈Rd

Here, $x={\left(x_{1},\ldots ,x_{d}\right)}^{T}$ denotes a decision vector in a *d*-dimensional search space, and F(x) contains m≥2 objective functions. The function $g_{j}\left(x\right)$ describes the *j*th inequality constraint [9]. Since equality constraints can be represented by relaxed inequality forms, the following discussion focuses on inequality-constrained problems.

The overall degree of infeasibility of a candidate is measured by accumulating only the positive parts of its constraint functions:

(2) $$\mathrm{CV}\left(x\right)=\sum_{j=1}^{n}\max\left(0,g_{j}\left(x\right)\right).$$

Accordingly, x is feasible when CV(x)=0 and infeasible otherwise. Dominance is defined only among feasible candidates. Given two feasible solutions $x_{1}$ and $x_{2}$, $x_{1}$ dominates $x_{2}$, written as $x_{1}⪯x_{2}$, when it is no worse in every objective and is strictly better in at least one objective. A feasible solution $x^{*}$ is Pareto optimal if no other feasible candidate dominates it. All such solutions constitute the constrained Pareto set (PS), and their objective vectors form the constrained Pareto front (PF).

The development of constrained multiobjective evolutionary algorithms (CMOEAs) has gradually shifted from relying on isolated constraint-handling mechanisms to organizing the search through multiple stages, interacting populations, and auxiliary tasks, so that objective progress and feasibility pressure can be coordinated more flexibly. Early methods typically adjust constraint tolerance or selection preferences dynamically, gradually shifting the search from objective exploration toward stronger feasibility pressure. For example, the ϵ-constraint mechanism adaptively controls the tolerance level to alleviate search blockage caused by strict feasibility rules [10]. MOEA/D-DAE switches between constraint-handling techniques according to the evolutionary state [11]. Multistage methods subsequently introduced explicit search phases. PPS weakens constraints initially to explore promising objective regions and restores constraint pressure later to approach the constrained PF [12]. These methods indicate that appropriate use of infeasible solutions can substantially improve global search under complex constraints. However, determining when and to what extent constraints should be relaxed remains critical to algorithm robustness.

Recent studies have increasingly focused on cooperation among multiple search processes. Coevolutionary frameworks maintain populations with different constraint preferences. One population pursues feasible convergence, while another population or auxiliary task explores regions that are difficult to reach under strict constraints [13]. CMOEA-CDT further constructs separate auxiliary tasks for convergence and diversity and improves the main-task search through knowledge transfer [14]. Dynamic constraint relaxation is used in DRCMO to improve the ability of the auxiliary search process to move across infeasible regions [15]. Another line of work focuses on the evaluation cost associated with maintaining auxiliary search. APSEA reduces or expands the auxiliary population in response to the evolutionary state [16], while RACMO evaluates the usefulness of auxiliary tasks to the main task and adjusts their resource shares through a competitive mechanism [17]. DPVAPS further regulates auxiliary-search resources by adapting the population structure according to the search state, whereas MGCMOEA allocates offspring between two populations according to multicriteria game-based payoffs [18,19]. These methods address different aspects of cooperative constrained search: DRCMO primarily adapts constraint relaxation, DPVAPS adjusts auxiliary-search resources through population adaptation, and MGCMOEA regulates offspring allocation through game-based performance evaluation. However, they do not jointly represent objective-wise search information as propagated vector signals, use these signals to determine individual constraint tolerance, and reuse the resulting search evidence for homeostatic offspring allocation. These studies show that CMOEA design has progressed from constructing constraint-handling rules alone toward jointly organizing search roles, exploiting infeasible information, and managing computational resources. Despite these advances, several interrelated issues on complex CMOPs require further investigation. First, excessive emphasis on feasibility can make the auxiliary population behave similarly to the main population, limiting its ability to cross infeasible barriers. Excessive constraint relaxation can instead retain many individuals with poor objective quality and divert the search from useful Pareto tradeoff regions. Second, many auxiliary-search mechanisms compress objective quality, constraint violation, and diversity into a scalar evaluation. Although convenient for ranking, this compression can obscure structural differences among objective directions and eventually concentrate the auxiliary population in a few compromise regions. Finally, multipopulation methods generally require additional function evaluations. A fixed resource ratio can waste evaluations when the effective contributions of the two populations vary across search stages. Conversely, allocation based too strongly on single-generation returns can rapidly deprive a temporarily weaker population of reproduction opportunities and reduce its ability to recover. Therefore, the remaining challenge is not only to relax constraints or redistribute search resources, but also to preserve objective-specific search directions while making constraint tolerance and resource allocation respond consistently to the same evolving search state.

To address these issues, this paper proposes the Multi-Morphogen Field-Guided Constrained Multiobjective Evolutionary Algorithm (MFGCMOEA). The algorithm maintains a main population and an auxiliary population that cooperate in parallel. The main population maintains strict constraint-based convergence pressure. The auxiliary population treats each objective as an independent morphogen channel and propagates local developmental signals according to the current relative relationships in the objective space. Rather than directly aggregating the objectives into one score, the propagated multichannel signals are retained as vector-valued developmental phenotypes to preserve different Pareto tradeoff directions. Morphogen intensities also adaptively determine developmental buffers. Additional stress is imposed only when a constraint disturbance exceeds the corresponding buffer, allowing infeasible solutions with objective potential to remain useful. This mechanism differs from direct dynamic-relaxation strategies such as DRCMO in that the relaxation level is not independently scheduled or adjusted as a separate constraint-handling quantity. Instead, the admissible constraint disturbance is derived from the same objective-wise propagated signals used to describe the developmental state of each candidate. Auxiliary environmental selection then performs nondominated filtering in the multidimensional phenotype space. Local contact inhibition regulates redundant occupancy only on the critical front, balancing search directions and solution distribution.

A homeostatic developmental resource allocation mechanism is further designed. It combines the quality of the two offspring sets in the developmental phenotype space with their actual recruitment rates into the new auxiliary population as search evidence. The mean evidence of the two populations determines a symmetric basal growth term. Under an unchanged total offspring budget per generation, the mechanism dynamically adjusts the offspring counts according to search contributions and prevents a population from being reduced prematurely because of short-term underperformance. Unlike DPVAPS, which changes auxiliary-search resources through population adaptation, and MGCMOEA, which determines offspring allocation from multicriteria game payoffs, MFGCMOEA keeps the environmental population sizes unchanged and redistributes the offspring budget using developmental quality and realized recruitment evidence. The additional basal growth term explicitly limits excessive resource polarization and preserves the recovery opportunity of a temporarily weak search process. MFGCMOEA thus integrates multiobjective structure preservation, constraint relaxation, and resource regulation within one dual-population framework. The auxiliary population can continuously explore promising routes without reproducing the main population’s feasibility pressure, while adaptive allocation improves the use of a limited evaluation budget.

The main contributions of this paper are summarized as follows:

1. A multi-morphogen developmental field-based auxiliary selection mechanism is proposed to preserve objective-specific search directions while adaptively relaxing constraints. Each objective is represented by an independent morphogen channel, and neighborhood propagation in objective-rank space forms a vector-valued developmental phenotype. The propagated signals further determine individual constraint buffers, allowing promising infeasible solutions to remain competitive without reducing the multiobjective information to a single scalar criterion.
2. A homeostatic developmental resource allocation mechanism is proposed to dynamically coordinate the two search processes. Offspring developmental quality and actual recruitment are combined as resource evidence, while a symmetric basal growth term prevents excessive allocation polarization. Under a fixed total offspring budget, the mechanism adapts the offspring numbers according to current search contributions while preserving the recovery capability of temporarily weaker populations.
3. The experimental results over four benchmark suites and 47 test problems place MFGCMOEA first overall for both IGD and HV among the nine compared methods. Its search process remains effective across different types of constraint landscapes, as reflected in the convergence and distribution results. The statistical analysis and ablation experiments further verify that the observed performance gains are associated with the proposed algorithmic components.

The rest of the manuscript proceeds from methodological background to algorithm design and experimental validation. Related CMOEA research is discussed in Section 2. Section 3 then develops MFGCMOEA and analyzes its computational complexity. The experimental setup is given in Section 4, while Section 5 evaluates the algorithm from comparative, convergence, diversity, statistical, and ablation perspectives. The main conclusions and future research directions are presented in Section 6.

## 2. Related Work on CMOEAs

The central difficulty in constrained multiobjective evolutionary optimization is not merely to find feasible solutions, but to continually adjust selection pressure among objective convergence, constraint satisfaction, and solution distribution. Complex constraints often produce narrow, disconnected, or mutually isolated feasible regions. Strict feasibility-first rules can consistently drive a population toward feasibility, but they may prematurely discard infeasible search directions with favorable objective potential. Excessive constraint relaxation can instead consume resources in unproductive regions. Existing CMOEAs can therefore be grouped into four categories according to how they organize search pressure: progressive evolution, multipopulation cooperation, multistage search, and hybrid models. These categories are not mutually exclusive. They address objective–constraint conflicts through constraint-pressure regulation, search–role decoupling, temporal search partitioning, and coordination among multiple mechanisms, respectively.

### 2.1. Progressive Evolution Methods

Progressive evolution methods usually maintain one primary search process and continuously adjust constraint handling and environmental selection according to the generation, population state, or local structure. They avoid maintaining multiple additional full populations and therefore have relatively compact computational frameworks.

Several widely used constraint-handling principles were established before the recent development of specialized CMOEAs. Deb et al. [20] incorporated feasibility-based constrained dominance into NSGA-II, allowing feasible solutions to be preferred without introducing explicit penalty coefficients. Takahama and Sakai [10,21] developed ϵ-constrained comparison mechanisms that permit mildly infeasible solutions to compete according to objective quality. These studies established the basic principle that feasibility pressure need not remain uniformly strict throughout the evolutionary process. Wang et al. [22] introduced decomposition into constrained multiobjective optimization. Weight directions transform the original problem into cooperative subproblems, whose updates consider both objective improvement and constraint status. This structure helps maintain different objective directions. However, when large infeasible regions block certain weight directions, the same update rule must still balance progress along the objective direction against movement toward feasibility. Yu et al. [23] proposed dynamic selection preferences that gradually shift selection pressure from early objective optimization toward constraint satisfaction. Two differential operators separately strengthen exploitation and exploration. Compared with a fixed constraint priority, this continuous adjustment reduces stagnation caused by excessive early feasibility pressure. Nevertheless, the search behavior still depends on how the evolutionary process represents the relative priorities of objectives and constraints. To make constraint regulation more dependent on the population state, Gu et al. [24] developed an adaptive constraint regulation mechanism based on nearest-neighbor relationships. The local neighborhood size is adjusted dynamically, allowing some competitive infeasible solutions to continue evolving at particular stages. By linking constraint tolerance to the current solution distribution, the method reduces the dependence of a fixed ϵ threshold on problem scale and feasible-region structure. The quality of local regulation nevertheless remains dependent on the sampled distribution. Zuo et al. [25] constructed reinforcement learning states from population feasibility and selected search operators online, replacing a predefined schedule with state feedback. Operator preferences can thus change with the search state, although state discretization and delayed rewards affect the accuracy of contribution assessment. Zuo et al. [26] further learned a global principal direction from the current population and formed tributary search directions through single-constraint ranking. Feasibility progress and objective search are incorporated into the same iteration, avoiding explicit stage switching. Overall, progressive evolution methods adapt search pressure with limited structural overhead. However, their different search objectives still rely on one population and one survival channel. Preserving substantially different search information is therefore difficult when strict feasible convergence and exploration across infeasible regions must coexist over an extended period.

### 2.2. Multipopulation Methods

Multipopulation methods assign relatively independent populations to different search tasks, so that feasible convergence and infeasible-region exploration need not share exactly the same environmental selection pressure.

The bidirectional coevolution method proposed by Liu et al. [27] employs two populations with different search preferences. Angle-based selection and restricted mating enable bidirectional information exchange between constrained search and objective exploration. This role separation reduces the probability that high-quality infeasible solutions are immediately eliminated by strict feasibility rules. However, maintaining fixed population sizes introduces additional evaluation overhead when their contributions vary across stages. To address this issue, Qiao et al. [28] incorporated dynamic constraint handling and resource allocation into a dual-population framework. Subsequent offspring counts are adjusted according to the effective proportions of the two offspring sets in environmental selection. Computational resources can thus vary with search contributions. This mechanism jointly considers search roles and evaluation budgets, but its evidence is mainly obtained from current selection outcomes and is consequently susceptible to single-generation fluctuations. As the role of infeasible solutions has become better understood, multipopulation methods have moved beyond the binary choice of considering or ignoring constraints toward more fine-grained search roles. Liu et al. [29] divided infeasible nondominated solutions into inner and outer populations, which provide escape and expansion forces, respectively. These forces enable the search to leave local feasible regions and expand toward unexplored feasible regions. Liu et al. [30] used constraint–Pareto dominance and several external archives with different responsibilities to separate forward exploration, diversity enhancement, and feasibility exploitation. This separation prevents a single feasibility criterion from obscuring infeasible solutions with favorable objective quality. Yu et al. [31] further developed a three-population framework combining cooperation and competition. Different auxiliary populations explore regions near the unconstrained front and between the unconstrained and constrained fronts. Resources are dynamically allocated according to their usefulness to the main search. Recent dual-population methods have further emphasized constraint relaxation and computational resource management. DPVAPS [18] adjusts auxiliary-search resources through population-size adaptation, thereby reducing unnecessary auxiliary evaluations as the search proceeds. DRCMO [15] focuses on dynamic constraint relaxation to enhance the auxiliary population’s ability to traverse infeasible regions. MGCMOEA [19] evaluates the search performance of two populations through multicriteria game-based payoffs and uses the resulting information to allocate offspring resources. Although these methods improve different aspects of cooperative constrained search, their objective representation, constraint regulation, and resource allocation are largely designed as separate control components. Such designs substantially improve adaptability to complex feasible regions, but additional search roles impose greater demands on information exchange, resource allocation, and contribution assessment. Auxiliary tasks that do not match the problem structure, or inaccurate short-term contribution estimates, can turn additional populations into unproductive computational overhead.

### 2.3. Multistage Methods

Multistage methods separate conflicting search objectives in time by assigning different priorities to objective optimization, feasibility progress, and diversity maintenance at different evolutionary stages.

In CTSEA, Ming et al. [32] employed a two-phase search strategy that separates objective exploration from constrained convergence. During the initial phase, constraint information is temporarily disregarded so that the population can approach the unconstrained PF, with informative search states retained in an archive for later use. After the transition, constraint-aware selection guides the population toward the constrained PF. The unconstrained front can provide strong objective-direction information. However, when it differs substantially from the constrained front, the accumulated information may not directly support subsequent search. Dong et al. [33] jointly assessed convergence, diversity, and feasibility in the first stage, then prioritized feasibility and used SPEA2-type evaluation to accelerate convergence in the second stage. This avoids defining stage objectives through a single indicator. Wang et al. [34] further introduced three stages for convergence enhancement, distribution expansion, and uniform search on the constrained front. Angle dominance and minimum-neighborhood dominance are used in the latter two stages to better align environmental selection with the stage objectives. Recent multistage strategies have incorporated problem-structure identification and variable analysis to reduce the limitations of fixed stage rules across different CMOPs. Ma et al. [35] first used dominant-constraint analysis to locate potentially feasible regions, then identified constraint-sensitive variables and applied different updates to different variable types. Stage transitions are thereby connected to the effects of decision variables on constraints. Zhou et al. [36] continuously updated information about the unconstrained and constrained fronts through archives. Online problem identification determines their relationship, and different constraint-handling methods are applied to different problem types. Compared with generation-based switching, these approaches make stage strategies more dependent on the current problem state. Their performance, however, depends on the accuracy of transition criteria, problem classification, or structural identification. Delayed or incorrect decisions can maintain an earlier stage’s pressure for too long or prematurely suppress infeasible directions that remain useful.

### 2.4. Hybrid Model-Based Methods

Hybrid models combine multipopulation, multistage, multitask, decomposition, or resource-management mechanisms to exploit their complementary strengths.

Liang et al. [37] explicitly used the relationship between the unconstrained and constrained PFs. The problem type is learned early, and a corresponding search strategy is used during subsequent coevolution. Auxiliary information is therefore exploited without assuming that the unconstrained front is always beneficial. Xie et al. [38] integrated a dual-population framework with a two-stage search process. The pulling population initially investigates the unconstrained front and subsequently redirects its search toward the constrained PF. Information exchange between the two populations is reinforced through dynamic cooperation, boundary-oriented sampling, and adaptive environmental selection. Although this type of stage-dependent design allows auxiliary roles to vary during evolution, its effectiveness remains sensitive to the timing of module transitions and the quality of transferred information. Hybrid CMOEAs have also increasingly incorporated explicit resource management and constraint decoupling. In BPRRA, Qu et al. [39] employ two populations to investigate distinct promising boundary regions during the early stage. The later stage reallocates the evaluation budget according to the fraction of offspring admitted to the archive, thereby assigning more computational effort to search processes that make larger current contributions. Li et al. [40] pursue a different direction by decomposing a problem with multiple constraints into cloned subtasks that follow different constraint-processing orders. Online knowledge exchange among these subtasks is then used to weaken the coupling among constraints. This decomposition creates multiple search trajectories for handling complex constraint interactions, although the coordination burden increases as additional constraints lead to more subtasks. Hou et al. [41] integrated dual populations, two stages, and M2M decomposition into one framework. An adaptive ϵ mechanism regulates constraint pressure in the main population and strengthens cooperation among search directions across stages. Overall, hybrid models offer considerable adaptability and information utilization, but simultaneous mechanisms also increase internal coupling. Tasks, populations, or stages often require additional state assessment, contribution evaluation, or parameter coordination. When these control quantities do not reflect the actual search state, the complex structure can itself become a resource burden. As summarized in Table 1, the main distinction of MFGCMOEA does not lie in the dual-population structure itself. The auxiliary population preserves objective-wise information through multiple morphogen channels and propagates these signals in objective-rank space. The resulting developmental state is further used to construct individual constraint buffers, rather than regulating constraint tolerance independently of objective information. The same developmental information is subsequently reused when estimating offspring quality for resource allocation. In addition, the homeostatic basal-growth term limits excessive resource polarization and retains recovery opportunities for a temporarily weaker search process. Therefore, rank-space propagation and homeostatic regulation respectively address objective-structure preservation with adaptive constraint tolerance and stable resource adjustment.

In summary, progressive evolution methods provide compact structures and continuous pressure regulation, but a single population struggles to sustain both strict feasible convergence and deep infeasible exploration. Multipopulation methods explicitly decouple search roles, yet must also address information redundancy and evaluation-budget allocation. Multistage methods separate search objectives in time, but uncertainty in stage boundaries and state identification can cause useful information to be discarded prematurely. Hybrid models improve adaptability while increasing coupling among evaluation criteria, tasks, and control mechanisms. Auxiliary search also often compresses objective quality, constraint violation, and distribution information into a few composite indicators. When objectives have different effective search directions, such compression can weaken the Pareto tradeoff structure. Resource allocation based only on immediate contributions can additionally deprive temporarily weaker but potentially useful processes of reproduction opportunities. The above comparison further indicates that objective representation, constraint tolerance, and resource regulation are commonly treated as relatively independent components. Their internal information is seldom reused consistently across auxiliary selection and resource allocation. These observations motivate an approach that preserves the independent structure of objective directions in the auxiliary population, adaptively determines constraint tolerance from the current search state, and allocates resources using both offspring quality and actual recruitment. The dual-population structure remains explicit, and the total evaluation budget remains fixed. Accordingly, the following algorithm uses multi-morphogen developmental fields for auxiliary environmental selection and homeostatic developmental resource allocation to respond to search contributions while limiting excessive resource polarization.

## 3. Proposed Algorithm

### 3.1. Algorithm Framework

**Figure 1:**
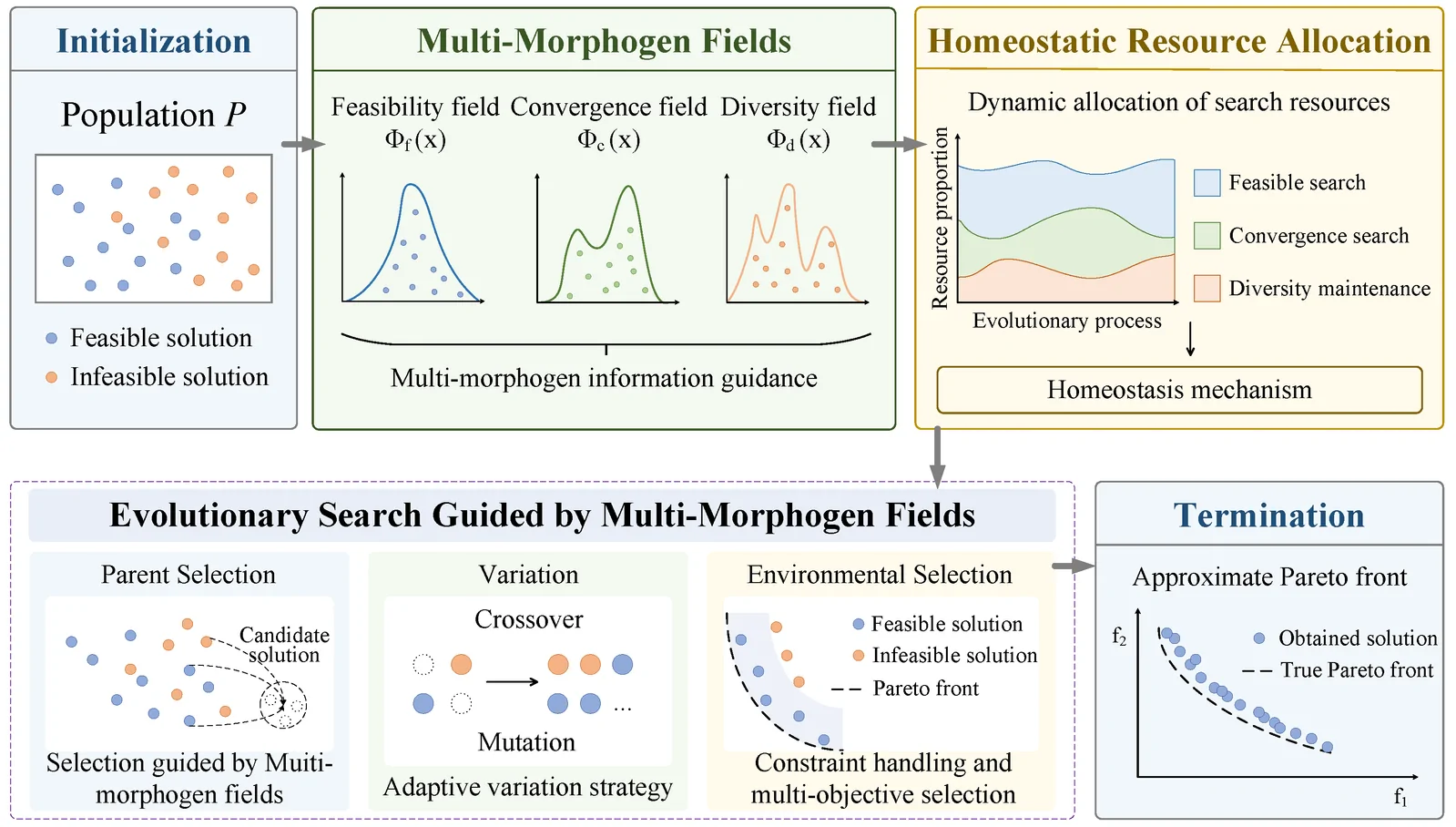
Schematic diagram of the MFGCMOEA framework.

MFGCMOEA adopts a parallel cooperative framework with a main population and an auxiliary population. The main population continuously approaches the feasible PF under strict constraint-based competition. Rather than reproducing the same feasibility pressure, the auxiliary population treats each objective as an independent morphogen channel. Local signal propagation forms multidimensional developmental phenotypes, while constraint buffers adaptively determined by morphogen intensities preserve infeasible search directions with objective potential. To avoid clustering of the auxiliary population caused by compressing multiple objectives into one scalar, environmental selection always performs nondominated filtering in the multidimensional phenotype space. Local contact inhibition regulates distribution only on the critical front. The algorithm estimates search contributions from the developmental quality of the two offspring sets and their actual recruitment rates into the auxiliary population. A homeostatic basal growth term, jointly determined by the current contributions of both populations, is used to adjust next-generation offspring counts under a fixed total evaluation budget. The main population thus provides reliable feasible convergence pressure, the auxiliary population preserves the ability to explore promising routes outside the feasible region, and resource allocation adjusts their computational effort using current search evidence. Algorithm 1 presents the overall procedure. The schematic diagram of the MFGCMOEA framework is shown in Figure 1.

Lines 1–2 of Algorithm 1 initialize two identical populations and compute constraint-aware search priorities for the main population. Lines 4–5 use two differential evolution operators to generate offspring for the main and auxiliary search directions. Lines 6–7 update the main population using both populations and all offspring, maintaining a stable drive toward feasibility and objective convergence. Lines 8–9 construct a multi-morphogen developmental field on the union of the updated main population, the previous auxiliary population, and both offspring sets to obtain the new auxiliary population. Finally, line 10 reuses the propagation matrix, developmental front levels, and individual recruitment indicators produced by auxiliary selection to allocate $N_{1}$ and $N_{2}$ for the next generation. The total offspring budget remains 2N per generation. These three shared quantities are denoted by $\Theta^{t}=\left\{W^{t},\nu^{t},b^{t}\right\}$, where $\nu_{i}^{t}$ is the developmental front index of candidate *i* and $b_{i}^{t}$ indicates whether it enters the new auxiliary population. The two proposed modules therefore determine which auxiliary search states to retain and which search process should receive more evaluations, respectively, while sharing the same current search evidence.
**Algorithm 1** Overall framework of MFGCMOEA**Require:** Population size *N*; maximum number of function evaluations 
**Ensure:** Main population $P_{1}$
  1:Randomly initialize $P_{1}^{0}$; set $P_{2}^{0}=P_{1}^{0}$ and $N_{1}=N_{2}=N$  2:Compute the constraint-dominance fitness of $P_{1}^{0}$  3:**while** the termination condition is not met **do**  4:    Generate $O_{1}^{t}$ from $P_{1}^{t}$, $P_{2}^{t}$, and the current fitness, with $|O_{1}^{t}|=N_{1}$  5:    Generate $O_{2}^{t}$ from $P_{1}^{t}$ and $P_{2}^{t}$, with $|O_{2}^{t}|=N_{2}$  6:    Construct $R_{1}^{t}=P_{1}^{t}\cup P_{2}^{t}\cup O_{1}^{t}\cup O_{2}^{t}$  7:    Select $P_{1}^{t+1}$ from $R_{1}^{t}$ using constraint-dominance fitness and distance truncation  8:    Construct $R_{2}^{t}=P_{1}^{t+1}\cup P_{2}^{t}\cup O_{1}^{t}\cup O_{2}^{t}$  9:    $(P_{2}^{t+1},\Theta^{t})\leftarrow$ multi-morphogen developmental field-based auxiliary selection $\left(R_{2}^{t},N\right)$10:    $(N_{1},N_{2})\leftarrow$ homeostatic developmental resource allocation $\left(O_{1}^{t},O_{2}^{t},\Theta^{t},2N\right)$11:    t←t+112:**end while**


### 3.2. Multi-Morphogen Developmental Field-Based Auxiliary Selection

The auxiliary population is intended not to replicate the main population’s feasibility rules, but to retain promising objective-improvement directions when complex constraint boundaries obstruct the main search. Simply ignoring constraints can disperse auxiliary individuals along a few extreme objective directions. Compressing multiple objectives into one scalar score can instead attract different Pareto tradeoffs toward the same compromise criterion, causing clustering and weakening the directional differences needed by differential evolution. Each objective is therefore treated as an independent morphogen channel. Relative objective quality generates local activation signals, which propagate through neighborhoods defined by the current objective-space geometry. The resulting signals encode both intrinsic individual states and nearby promising search directions. Constraint information is not directly weighted with the objectives. Instead, it is transformed into a dimensionless relative disturbance. The propagated morphogen channels jointly determine each individual’s developmental buffering capacity, and additional stress arises only when the disturbance exceeds this buffer. Each individual retains an *M*-dimensional developmental phenotype on which nondominated sorting is performed, without first compressing competition among objectives. Distribution control is restricted to the last developmental front requiring truncation. Contact inhibition discourages redundant occupancy by local phenotypes that are highly similar to selected individuals. No fixed objective weights, constraint penalty coefficients, or neighborhood radii are specified. Algorithm 2 details the procedure.
**Algorithm 2** Multi-morphogen developmental field-based auxiliary selection**Require:** Candidate set $R_{2}={\left\{x_{i}\right\}}_{i=1}^{K}$; objective count *M*; constraint count *L*; population size *N*

**Ensure:** New auxiliary population $P_{2}$; developmental-field information Θ
  1:Compute the empirical rank $z_{im}$ for each objective and set $a_{im}=1-z_{im}$  2:Compute $d_{ij}$ in rank space and set *h* to the median positive distance  3:Compute propagation strengths $w_{ij}$ and row-normalized weights ${\hat{w}}_{ij}$  4:Propagate each objective-channel signal to obtain ${\bar{a}}_{im}$  5:Rank positive violations within each constraint to obtain relative disturbance $c_{i}$  6:Compute developmental buffer $\tau_{i}$ and residual stress $s_{i}$ from ${\left\{{\bar{a}}_{im}\right\}}_{m=1}^{M}$  7:Construct the multidimensional phenotype $y_{i}=\left(y_{i1},\ldots ,y_{iM}\right)$  8:Perform nondominated sorting on $\left\{y_{i}\right\}$, record front indices $\nu_{i}$, and retain complete fronts in order  9:Fill the remaining positions from the critical front using contact saturation until *N* individuals are selected; obtain recruitment indicators $b_{i}$10:Return $P_{2}$ and Θ={W,ν,b} for resource allocation


Let *K* be the auxiliary candidate-set size and $f_{im}$ the value of the *m*th minimization objective for individual *i*. To remove differences in objective scales, an empirical rank transformation is applied separately to each objective column:

(3) $$z_{im}=\frac{\mathrm{rank}(f_{im})}{K+1},\;a_{im}=1-z_{im},$$

where $\mathrm{rank}(f_{im})$ is the positional rank obtained by sorting the *m*th objective in ascending order, taking values in 1,…,K. Thus, $z_{im}\in \left(0,1\right)$, and a smaller objective value gives a larger initial morphogen activation $a_{im}$. Unlike direct aggregation into a scalar fitness, $a_{i}=\left(a_{i1},\ldots ,a_{iM}\right)$ preserves independent information from all objective channels. Because the rank is evaluated within the current candidate set, $z_{im}$ is a relative rather than an absolute objective representation. Consequently, the rank of an unchanged individual may vary when the composition of $R_{2}$ changes. This context dependence is intentional: it removes dependence on objective magnitudes and allows the developmental field to adapt to the current search distribution, although the resulting representation is not invariant to changes in candidate-set composition.

To establish morphogen propagation relationships, the Euclidean distance between individuals *i* and *j* in objective-rank space is defined as

(4) $$d_{ij}={∥z_{i}-z_{j}∥}_{2}.$$

Let $D_{+}=\left\{d_{ij}:d_{ij}>0\right\}$. When $D_{+}$ is nonempty, its current median adaptively determines the propagation scale, i.e., $h=\mathrm{median}(D_{+})$. If all rank vectors coincide, h=1 is used to keep the propagation kernel well defined; this value is only a numerical treatment of the degenerate case. The bounded propagation strength is then constructed as

(5) $$w_{ij}=\frac{1}{1+{\left(d_{ij}/h\right)}^{2}},\;w_{ii}=0,$$

and normalized by row as

(6) $${\hat{w}}_{ij}=\frac{w_{ij}}{\sum_{r=1}^{K}w_{ir}}.$$

The kernel forms a soft fully connected propagation graph: every nonidentical candidate has a nonzero influence, while the influence decreases continuously with rank-space distance. Hence, the method does not impose a hard neighborhood boundary or a preset number of neighbors. The median scale *h* adapts the decay to the current population geometry, so nearby candidates contribute more strongly than distant ones after row normalization. This design favors smooth information propagation, while its possible sensitivity to global signal mixing is examined experimentally in the revised study.

The neighborhood signal received by individual *i* in morphogen channel *m* is

(7) $$\ell_{im}=\sum_{j=1}^{K}{\hat{w}}_{ij}a_{jm},$$

The intrinsic and neighborhood signals are integrated using the following bounded union form:

(8) $${\bar{a}}_{im}=1-\left(1-a_{im}\right)\left(1-\ell_{im}\right).$$

Thus, ${\bar{a}}_{im}$ reflects both the individual’s quality on objective *m* and the developmental state of the same objective channel in its neighborhood. Its value remains in [0,1].

Constraint handling uses relative disturbances that are independent of objective scales. Let $v_{il}=\max\left\{0,g_{l}\left(x_{i}\right)\right\}$ denote the positive violation of constraint *l*. For individuals with $v_{il}>0$, ascending positional ranks are assigned only within the positive-violation set of that constraint. Dividing each rank by the number of violating individuals plus 1 gives $r_{il}^{c}$. Individuals satisfying the constraint are assigned $r_{il}^{c}=0$. The aggregate disturbance across all constraints is defined as

(9) $$c_{i}=\sqrt{\frac{1}{L}\sum_{l=1}^{L}{\left(r_{il}^{c}\right)}^{2}}.$$

This root-mean-square aggregation treats all constraints symmetrically and avoids introducing problem-dependent constraint weights. Let $A_{i}=\left\{l:r_{il}^{c}>0\right\}$ denote the set of constraints violated by candidate *i*, and let $k_{i}=\left|A_{i}\right|$. For $k_{i}>0$, Equation (9) can equivalently be written as

$$c_{i}=\sqrt{\frac{k_{i}}{L}}\sqrt{\frac{1}{k_{i}}\sum_{l\in A_{i}}{\left(r_{il}^{c}\right)}^{2}}.$$

Hence, the disturbance can be interpreted as the product of two terms: the square root of the proportion of violated constraints, $\sqrt{k_{i}/L}$, and the root-mean-square severity of their relative violation ranks. Increasing either the number of active violations or any active relative violation rank cannot decrease $c_{i}$ when the remaining quantities are fixed. At the same time, different violation patterns can produce the same disturbance if their squared relative-rank sums are equal. For example, one severe violation and several moderate violations are treated equivalently when they yield the same value of $\sum_{l}{\left(r_{il}^{c}\right)}^{2}$. Therefore, $c_{i}$ represents a normalized aggregate of violation breadth and relative severity rather than an absolute physical magnitude of constraint violation. The normalization by *L* further makes the measure depend on the proportion, rather than only the absolute number, of active constraints.

The propagated morphogen signals further determine the constraint disturbance that an individual can tolerate. The developmental buffer is defined as

(10) $$\tau_{i}=1-{\left[\prod_{m=1}^{M}\left(1-{\bar{a}}_{im}\right)\right]}^{1/M}.$$

A strong developmental signal in one or more objective channels increases $\tau_{i}$, allowing the individual to enter deeper infeasible regions while retaining objective potential. Accordingly, the buffer intentionally gives greater tolerance to candidates that exhibit strong developmental signals in at least part of the objective space. This tolerance does not imply unconditional survival of highly infeasible candidates. Their final competitiveness is still determined jointly by the residual stress, the channel-wise developmental deficits, and nondominated sorting in the resulting vector phenotype.

A constraint disturbance generates residual stress only after exceeding the buffer:

(11) $$s_{i}=\left\{\begin{array}{cc} 0, & c_{i}\leq \tau_{i},\\ \frac{c_{i}-\tau_{i}}{1-\tau_{i}}, & c_{i}>\tau_{i}. \end{array}\right.$$

The effective developmental phenotype in channel *m* is then given by

(12) $$y_{im}=1-{\bar{a}}_{im}+s_{i}.$$

The residual stress $s_{i}$ uniformly increases the developmental deficits across channels only when the buffer is insufficient. Infeasible individuals within the relaxation region are therefore not implicitly reranked by their raw violation magnitudes, whereas severely infeasible individuals are still moved toward worse developmental fronts. Thus, the mechanism does not remove constraint pressure; instead, it postpones that pressure only for candidates whose disturbance remains compatible with their current developmental state. The distribution of buffers and retained constraint disturbances, together with the contribution of retained infeasible solutions to later feasible search, is evaluated separately in the revised experiments.

The algorithm then performs nondominated sorting on $y_{i}=\left(y_{i1},\ldots ,y_{iM}\right)$ under minimization, denotes the front index of candidate *i* by $\nu_{i}$, and preferentially retains complete better fronts. If the last front cannot be retained in full, contact inhibition is applied only to this critical front. When complete fronts have already been retained, let S be the selected set. The saturation of an unselected critical-front individual *i* is

(13) $$\eta_{i}=\max_{j\in S}w_{ij}.$$

The individual with the smallest $\eta_{i}$ is selected first. Ties are broken by the smaller mean developmental deficit $M^{-1}\sum_{m}y_{im}$. If the critical front is the first front and S is empty, the individual with the smallest mean developmental deficit is selected initially. Subsequent individuals are selected by the saturation rule. After each addition, saturation is updated using its propagation strengths $w_{ij}$ to the remaining candidates. This process continues until the auxiliary population contains *N* individuals. Finally, $b_{i}=1$ indicates that candidate $x_{i}$ is recruited into the new auxiliary population; otherwise, $b_{i}=0$. Diversity therefore affects only boundary candidates at the same developmental level. It cannot promote an individual with poor objective quality across developmental fronts.

### 3.3. Homeostatic Developmental Resource Allocation

Assigning the same offspring count to both populations in every generation implicitly assumes that their search processes have equal marginal value throughout evolution. This assumption is generally difficult to justify in constrained multiobjective optimization. Before a feasible region is found, auxiliary exploration of infeasible routes may be more useful. Once a stable feasible front begins to form, strict convergence in the main population may become the principal driving force. Conversely, allocation based only on immediate returns in one generation can rapidly reduce the offspring count of a temporarily weaker population and further limit its recovery opportunities. The proposed mechanism directly reuses the developmental fronts and local propagation relationships produced by auxiliary selection. Offspring contributions are evaluated from two complementary perspectives: developmental front quality with a bounded local-density adjustment, and the proportion of offspring actually recruited into the new auxiliary population. A bounded union of these two quantities forms the current developmental evidence for each population. The mean evidence of the two populations then provides a symmetric homeostatic basal growth term. Resource proportions can thereby respond to current differences without becoming excessively polarized after one poor generation. Algorithm 3 describes this procedure.
**Algorithm 3** Homeostatic developmental resource allocation**Require:** Offspring sets $O_{1},O_{2}$; developmental front indices $\nu_{i}$; propagation matrix *W*; auxiliary selection indicators $b_{i}$; total budget 2N

**Ensure:** Next-generation offspring counts $N_{1},N_{2}$
  1:Compute candidate front quality $q_{i}$ from the current maximum front index  2:Compute local propagation density and convert it to empirical rank $\rho_{i}$  3:Compute density-adjusted effective developmental quality $e_{i}$  4:**for** k∈{1,2} **do**  5:    Compute quality evidence $Q_{k}$ and recruitment evidence $R_{k}^{\mathrm{rec}}$ for $O_{k}$  6:    Compute combined developmental evidence $\Gamma_{k}$  7:**end for**  8:Compute the homeostatic basal growth term $B=(\Gamma_{1}+\Gamma_{2})/2$  9:If $\Gamma_{1}+\Gamma_{2}=0$, set $\pi_{1}=\pi_{2}=1/2$; otherwise, set $G_{k}=\Gamma_{k}+B$ and normalize $G_{k}$ to obtain $\pi_{k}$10:Allocate the 2N offspring positions to integer counts $N_{1},N_{2}$ by the largest-remainder method11:Return $N_{1},N_{2}$


Let $\nu_{\max}$ be the largest finite developmental front index obtained during auxiliary selection, and let $\nu_{i}$ be the front index of candidate $x_{i}$. Front levels are mapped to

(14) $$q_{i}=\frac{\nu_{\max}+1-\nu_{i}}{\nu_{\max}},$$

The first front has the highest quality, and $q_{i}$ decreases for deeper fronts. Meanwhile, the propagation matrix from the preceding subsection gives the local propagation density

(15) $$\delta_{i}^{\mathrm{loc}}=\frac{1}{K}\sum_{j=1}^{K}w_{ij},$$

The normalized ascending positional rank of $\delta_{i}^{\mathrm{loc}}$ is denoted by $\rho_{i}\in \left(0,1\right)$. Density serves only as a bounded secondary regulator. Effective developmental quality is defined as

(16) $$e_{i}=\frac{q_{i}}{1+\rho_{i}\left(1-q_{i}\right)}.$$

When $q_{i}$ is close to 1, local density has only a limited effect on high-quality offspring. Crowding further reduces resource evidence primarily for individuals on deeper developmental fronts, preventing sparsity alone from producing an excessively high evaluation. A special case occurs when all candidates belong to a single developmental front, i.e., $\nu_{\max}=1$. In this case, $q_{i}=1$ and consequently $e_{i}=1$ for every candidate, so the density term intentionally loses its discriminative effect. Because no front-level difference is available to distinguish the two search processes, the resulting quality evidence becomes neutral rather than introducing an artificial preference from density alone. Under the subsequent evidence aggregation, both populations therefore receive equal resource shares in this degenerate case. The frequency with which this situation occurs during evolution is examined in the experimental analysis.

For offspring set $O_{k}$ generated by search process *k*, quality evidence is the root mean square of effective developmental quality:

(17) $$Q_{k}=\sqrt{\frac{1}{|O_{k}|}\sum_{x_{i}\in O_{k}}e_{i}^{2}},\;k\in \left\{1,2\right\}.$$

Let $b_{i}=1$ indicate that candidate $x_{i}$ enters the new auxiliary population, and let $b_{i}=0$ otherwise. Actual recruitment evidence is defined as

(18) $$R_{k}^{\mathrm{rec}}=\frac{1}{|O_{k}|}\sum_{x_{i}\in O_{k}}b_{i}.$$

The quantity $Q_{k}$ reflects the overall developmental levels of the offspring, whereas $R_{k}^{\mathrm{rec}}$ directly measures their contribution to the current auxiliary-population update. The recruitment rate is therefore used as an immediate search-state indicator rather than as a direct estimate of final PF improvement. An offspring can be recruited because it preserves a useful developmental direction even if its benefit to the feasible main population is not realized in the same generation. For this reason, $R_{k}^{\mathrm{rec}}$ is not used alone, but is combined with developmental quality $Q_{k}$. The relationship between this recruitment evidence and subsequent feasible-search progress is evaluated separately in the experimental analysis. The two quantities are combined using a bounded union form:

(19) $$\Gamma_{k}=1-\left(1-Q_{k}\right)\left(1-R_{k}^{\mathrm{rec}}\right).$$

This form does not require manually specified weights. A population receives a corresponding positive developmental signal when either its quality evidence or actual recruitment evidence is high.

To reduce resource polarization caused by instantaneous evidence, a symmetric homeostatic basal growth term is constructed from the current evidence of both populations:

(20) $$B=\frac{\Gamma_{1}+\Gamma_{2}}{2},\;G_{k}=\Gamma_{k}+B.$$

The resource shares are given by

(21) $$\pi_{k}=\frac{G_{k}}{G_{1}+G_{2}},\;k\in \left\{1,2\right\},$$

and satisfy $\pi_{1}+\pi_{2}=1$. When $\Gamma_{1}+\Gamma_{2}>0$, substituting Equation (20) into Equation (21) gives

$$\pi_{k}=\frac{1}{4}+\frac{\Gamma_{k}}{2(\Gamma_{1}+\Gamma_{2})},$$

and hence

$$\frac{1}{4}\leq \pi_{k}\leq \frac{3}{4}.$$

Therefore, the homeostatic term allows the better-performing process to receive up to three times the offspring resources of the weaker process while preventing either population from being starved by short-term evidence. This bounded asymmetry is consistent with the purpose of dynamic allocation: it remains responsive to contribution differences while preserving the possibility of recovery. If $\Gamma_{1}=\Gamma_{2}=0$, no positive evidence is available from either search process and the implementation assigns the neutral allocation $\pi_{1}=\pi_{2}=1/2$, avoiding the undefined zero-denominator case.

With the total budget fixed at 2N, continuous offspring allocations $2N\pi_{k}$ are first computed and rounded down. The remaining positions are then assigned in descending order of fractional remainders, yielding $N_{1}$ and $N_{2}$ for the next generation. If integer allocation gives zero to one population, one minimal reproduction opportunity is retained. This implementation treatment prevents complete loss of offspring-generation capability at a discrete boundary; it is not a separate contribution of the allocation model.

### 3.4. Search Operators

The two populations have different search roles and therefore use different but coupled differential evolution updates. The main population must continually exploit good constraint-aware solutions while incorporating the directions preserved by the auxiliary population. A *p*-best-guided update is used for this purpose. The auxiliary population instead emphasizes expansion around base points supplied by the main population using its own difference vectors, and therefore uses a rand/1 update. Both operators select parents across populations. Infeasible directions retained by the auxiliary population can enter the main search, while convergence information from the main population supplies base points for auxiliary search. The following equations describe the differential update and crossover stages. Both offspring types subsequently undergo the same boundary repair and polynomial mutation.

For the first offspring type, let $x_{r_{0}}^{2,t}$ be a base vector from auxiliary population $P_{2}^{t}$, $x_{p}^{1,t}$ a *p*-best vector randomly selected from the best pN individuals in the main population by fitness, $x_{r_{1}}^{1,t}$ a random main-population vector, and $x_{r_{2}}^{2,t}$ a random auxiliary-population vector. The differential mutant in dimension *j* is first constructed as

(22) $$v_{i,j}^{1,t+1}=x_{r_{0},j}^{2,t}+F_{i}\left[x_{p,j}^{1,t}-x_{r_{0},j}^{2,t}+x_{r_{1},j}^{1,t}-x_{r_{2},j}^{2,t}\right],$$

The trial vector is then obtained using a crossover mask:

(23) $${\tilde{x}}_{i,j}^{1,t+1}=\left\{\begin{array}{cc} v_{i,j}^{1,t+1}, & \xi_{i,j}<CR_{i},\\ x_{r_{0},j}^{2,t}, & \mathrm{otherwise}. \end{array}\right.$$

where $\xi_{i,j}$ is uniformly distributed on [0,1], and $F_{i}$ and $CR_{i}$ are the scaling factor and crossover probability for the corresponding offspring. The update combines a directed difference toward a good main-population solution with a cross-population difference.

The second offspring type uses a randomly selected main-population individual $x_{r_{0}}^{1,t}$ as its base vector. Two random auxiliary-population individuals provide the difference vector. The update in dimension *j* is

(24) $${\tilde{x}}_{i,j}^{2,t+1}=\left\{\begin{array}{cc} x_{r_{0},j}^{1,t}+F_{i}\left(x_{r_{1},j}^{2,t}-x_{r_{2},j}^{2,t}\right),\; & \xi_{i,j}<CR_{i},\\ x_{r_{0},j}^{1,t}, & \mathrm{otherwise}. \end{array}\right.$$

where both $x_{r_{1}}^{2,t}$ and $x_{r_{2}}^{2,t}$ are taken from the auxiliary population. This operator uses a main-population state as the search base while preserving the internal difference structure of the auxiliary population. Auxiliary directions can therefore be continued without entirely departing from the current convergence region.

In the implementation, p=0.1, and the *p*-best set contains the max{1,⌊0.1N⌋} main-population individuals with the smallest current fitness values. Each offspring independently samples $F_{i}$ from {0.6,0.8,1.0} and $CR_{i}$ from {0.1,0.2,1.0}. After differential crossover, out-of-bound variables are clipped to their respective lower or upper bounds, followed by polynomial mutation. The mutation control parameter is 1 and the distribution index is 20, giving a per-variable mutation probability of 1/D, where *D* is the decision-space dimension. These parameter settings and operator forms follow the underlying differential search framework. The proposed innovations concern auxiliary environmental selection and offspring resource regulation [9].

### 3.5. Complexity Analysis

The combined candidate-set size is of the same order as *N* in each generation. The cost of the two differential operators is linear in the total offspring count and *D*. Objective and constraint sorting in auxiliary selection has linearithmic cost. Pairwise distances, the propagation matrix, developmental-field nondominated sorting, and critical-front contact inhibition require at most quadratic time for fixed *M* and *L*. Resource allocation reuses the propagation matrix and front information, and its additional computation is also at most quadratic. Constraint-dominance comparisons and objective distances in the main population are predominantly quadratic, while row-wise nearest-neighbor sorting incurs quadratic-logarithmic cost. If too many candidates have fitness below 1, sequential SPEA2-type distance truncation is triggered. Repeated sorting of the remaining distance matrix can require $O(N^{3}\log N)$ time in the worst case. The worst-case per-generation complexity of the current implementation is therefore determined by this inherited main-population truncation procedure. The proposed multi-morphogen selection and resource allocation do not increase this asymptotic upper bound. Consequently, MFGCMOEA introduces no additional order of complexity relative to dual-population CMOEAs using the same SPEA2-type truncation structure.

## 4. Experimental Settings

Eight representative algorithms are selected to assess MFGCMOEA against different constraint-handling and distribution-maintenance mechanisms. AGEMOEAII, ANSGAIII, and RVEA_*a*_ employ front geometry estimation, adaptive reference points, and reference-vector adaptation, respectively, and serve as baselines for objective-space distribution maintenance [42,43,44]. DPVAPS, DRCMO, and TriP organize search through auxiliary population size adjustment, dynamic constraint relaxation, and three-population cooperation, respectively. They are included to compare how different cooperative strategies exploit infeasible information [15,18,45]. FLI selects search operators using fitness landscape information, whereas TSTI restructures selection pressure through stage transitions. They are used to examine state-responsive and stage-based search [33,46].

Table 2 summarizes the parameter configurations adopted by all compared algorithms. The notation $N_{0}$ refers to the initial population size, while *D* denotes the dimensionality of the decision space. For algorithms using crossover and mutation, $p_{c}$ and $p_{m}$ represent the corresponding application probabilities, and $\eta_{c}$ and $\eta_{m}$ denote the distribution indices used in simulated binary crossover and polynomial mutations. The MFGCMOEA-specific settings include the candidate collections of differential scaling factors F and crossover rates $C_{R}$, together with *p*, which specifies the fraction of high-quality individuals considered during parent selection. Parameters not defined here follow the original notation of their respective algorithms.

Three complementary indicators are adopted to characterize the final solution quality from different perspectives. IGD evaluates how well the obtained feasible approximation represents the reference PF, HV measures the dominated region in objective space, and FSR reflects the degree of feasibility in the final population. Let P denote the population returned at the end of one run. The objective vectors of its feasible nondominated members form the set A, while R denotes the sampled reference PF associated with the test problem. The number of objectives is denoted by *M*.

(1) IGD. The inverted generational distance quantifies the discrepancy between the reference PF and the approximation obtained by the algorithm. For every reference point, the distance to its closest member in A is calculated and then averaged:
  (25) $$\mathrm{IGD}\left(A,R\right)=\frac{1}{\left|R\right|}\sum_{r\in R}\min_{a\in A}{∥r-a∥}_{2}.$$
  Here, |R| is the cardinality of the reference set and ${∥\cdot ∥}_{2}$ is the Euclidean norm. The same reference PF is used for every algorithm on a given problem. Hence, lower IGD values indicate that the returned approximation lies closer to, and more adequately represents, the reference front. The computation uses the reference objective values supplied by PlatEMO and does not introduce algorithm-dependent normalization [44].
(2) HV. Hypervolume evaluates the extent of objective space jointly dominated by the obtained approximation with respect to a prescribed reference point [47]. In the present experiments, HV is evaluated using the common metric routine implemented in PlatEMO. Consequently, all algorithms tested on the same benchmark problem share identical normalization information and the same HV reference point.
  Let $\tilde{A}$ be the normalized objective-vector set generated by the platform and let $r^{\mathrm{HV}}$ be the corresponding reference point. The metric is computed as
  (26) $$\mathrm{HV}\left(A\right)={\mathrm{Vol}}_{M}\left(\underset{\tilde{a}\in \tilde{A}}{⋃}\prod_{m=1}^{M}\left[{\tilde{a}}_{m},r_{m}^{\mathrm{HV}}\right]\right),$$
  where ${\mathrm{Vol}}_{M}$ denotes the volume in the *M*-dimensional objective space. Because the normalization scheme and reference point are fixed at the problem level, the resulting HV values are evaluated on a common scale across all competing methods. Larger HV values correspond to a better joint outcome in convergence and objective-space coverage.
(3) FSR. The feasible solution ratio is used to characterize the feasibility level of the population returned by an algorithm. Rather than evaluating only the feasible nondominated subset, it considers every individual in P:
  (27) $$\mathrm{FSR}\left(P\right)=\frac{1}{\left|P\right|}\sum_{x\in P}I\left(\mathrm{CV}(x)=0\right),$$
  where I(·) equals 1 when its argument is true and 0 otherwise, and CV(x) follows the definition given previously. Thus, FSR is the fraction of feasible individuals in the final population and takes values in [0,1]. A larger value indicates stronger feasibility attainment. For repeated runs, the reported FSR is the average of the run-wise ratios and may be expressed as a percentage.

All numerical experiments are implemented in PlatEMO [44]. The test set consists of four benchmark suites, namely CF, DASCMOP, LIRCMOP, and MW, which contain 10, 9, 14, and 14 instances, respectively, for a total of 47 CMOPs [9,48,49,50]. Unless otherwise stated, every algorithm starts with a population size of 100 and is allowed at most maxFE = 100,000 function evaluations. Each algorithm–problem combination is repeated independently 30 times. The experiments were executed in MATLAB R2022b under 64-bit Windows 10 Pro for Workstations (version 22H2), using an AMD Ryzen 9 9950X 16-Core Processor operating at 4.30 GHz and 64 GB of RAM. Final IGD and HV values are summarized by their sample means and standard deviations, while FSR is used to complement these two indicators with feasibility information. A run that fails to produce any feasible solution is assigned NaN for both IGD and HV. For a given algorithm and problem, numerical mean and standard-deviation values are reported only when all 30 runs are feasible. Hence, a finite IGD or HV entry implies successful feasibility discovery in every run. If NaN is shown instead, the percentage reported in parentheses denotes the fraction of runs that obtained feasible solutions.

Pairwise performance differences are examined with the Wilcoxon test at the 0.05 significance level, with MFGCMOEA used as the control method throughout the result tables. In addition to these problem-wise comparisons, the overall differences across benchmark problems are assessed using the Friedman test, followed by Holm-adjusted post-hoc comparisons to control for multiple testing. The symbols “+”, “−”, and “=” attached to a competitor indicate that MFGCMOEA performs significantly better, significantly worse, or shows no significant difference, respectively. Lower IGD values are preferred, whereas larger HV and FSR values indicate better performance. The “+/−/=” summary at the bottom of each table counts the corresponding outcomes within that benchmark suite. Results ranked first for an indicator are highlighted in bold, and smaller mean-rank values indicate better aggregate performance over the complete test set.

## 5. Experimental Results and Analysis

### 5.1. CF Test Suite

The IGD and HV results on the CF test suite are reported in Table 3. The CF results first reveal differences between objective-space distribution mechanisms and constraint-handling capabilities. MFGCMOEA obtains a mean rank of 2.30 for both IGD and HV, ranks first on six of the ten problems, and significantly outperforms most competitors on seven to eight problems. By comparison, AGEMOEAII uses PF geometry estimation to improve environmental selection, whereas ANSGAIII mainly uses adaptive reference points to maintain solution-set coverage. Both mechanisms characterize objective-space structure effectively. However, when feasible regions restrict attainable objective directions, geometry or reference directions alone cannot continuously preserve individuals outside constraint boundaries that remain useful for subsequent search. The two algorithms consequently have poorer mean ranks on CF. MFGCMOEA treats each objective as an independent morphogen channel and performs nondominated filtering on multidimensional developmental phenotypes. Objective-direction information is therefore not reduced to one scalar before constraint handling. Developmental buffering imposes pressure only on constraint disturbances that exceed the capacity of the current signals. The auxiliary population can thus exploit some infeasible search information while retaining objective structure. CF8–CF10 also show that this advantage does not imply superiority on every front shape. On CF8 and CF9, reference-vector guidance and adaptation give RVEAa more competitive results. On CF1, FLI switches differential evolution strategies according to fitness landscape states and more rapidly exploits locally effective directions. TriP, which coordinates populations for the original, unconstrained, and constraint-relaxed problems in parallel, is also competitive on several problems. These results indicate that specialized reference directions or operator selection can be advantageous when the main difficulty concerns front coverage or local search efficiency at a particular stage. The advantage of MFGCMOEA lies more in a consistent balance across problems. It does not prescribe a single front model or stage-switching rule. Instead, multi-morphogen propagation, constraint buffering, and critical-front contact inhibition jointly regulate auxiliary search, yielding a lower overall rank across CF1–CF10.

### 5.2. DASCMOP Test Suite

The IGD and HV results on the DASCMOP test suite are reported in Table 4. DASCMOP simultaneously introduces feasibility, convergence, and diversity difficulties, placing stronger demands on the state adaptability of constraint handling. The advantages of MFGCMOEA are most concentrated on this suite. Its mean ranks are 1.11 for IGD and 1.56 for HV, and it ranks first in IGD on eight of the nine problems. Except for a few individual cases, MFGCMOEA significantly outperforms the competitors. DPVAPS and TriP are competitive dual- or multipopulation methods on this suite. DPVAPS reduces the auxiliary population as evolution proceeds, gradually releasing later evaluation resources to the main population. TriP assigns distinct roles to strictly constrained, unconstrained, and relaxed-constraint search. Both mechanisms alleviate search blockage caused by a single feasibility pressure, but their auxiliary-search intensity mainly follows predefined population roles or evolutionary progress. MFGCMOEA does not directly use the generation count to determine how much infeasible information the auxiliary population should retain. Its current multiobjective morphogen intensities generate individual developmental buffers. Different objective directions can therefore tolerate different constraint disturbances within the same generation, which is better suited to the simultaneous difficulties in DASCMOP. DRCMO also uses dual populations and dynamic constraint relaxation to help auxiliary search cross infeasible regions, but its mean IGD and HV ranks remain 5.22 and 5.00, respectively. This difference indicates that varying relaxation strength alone is insufficient to ensure effective auxiliary search. If a strong composite score still compresses relaxed individuals into a few directions, objective structure may not be adequately retained after crossing constraints. In addition to relaxation, MFGCMOEA preserves morphogen vectors and uses contact inhibition only on the critical developmental front. Allowing infeasible exploration and preserving different Pareto tradeoff directions are thus coordinated within the same selection process. TSTI’s two-stage, three-indicator mechanism is advantageous on individual problems such as DASCMOP8. This suggests that restructuring selection pressure by stage can be effective for particular combinations of difficulties. MFGCMOEA instead continually regulates pressure through a developmental field reconstructed in each generation, reducing dependence on stage boundaries and providing more consistent overall performance.

### 5.3. LIRCMOP Test Suite

The IGD and HV results on the LIRCMOP test suite are reported in Table 5. The main difficulty in LIRCMOP is that large infeasible regions block useful search routes. The suite therefore directly tests whether an auxiliary population can exploit infeasible information. MFGCMOEA achieves mean ranks of 1.43 for IGD and 1.50 for HV and ranks first on 12 problems for both indicators. The significance results also show advantages on almost all problems over methods including AGEMOEAII, ANSGAIII, and TSTI. For algorithms based on reference points or geometric modeling, uniform objective-space distribution mechanisms become fully effective only after the search reaches the corresponding regions. Large infeasible regions can disconnect these directions from the current feasible search trajectory. In MFGCMOEA, stronger objective signals provide larger developmental buffers, allowing the auxiliary population to cross infeasible regions along directions with potential objective value. The multichannel phenotype also prevents all individuals from converging toward the same compromise direction after crossing a barrier. TriP obtains the second-lowest mean rank on LIRCMOP. Its three-population structure explicitly retains unconstrained and relaxed-constraint search and is therefore also suited to large infeasible regions. FLI and DRCMO are competitive as well, improving local search and barrier crossing through landscape-driven operator switching and dynamic relaxation, respectively. However, TriP maintains three distinct roles simultaneously, FLI adapts mainly at the operator level, and DRCMO focuses on constraint-relaxation strength. MFGCMOEA integrates objective-direction preservation and relaxation into one developmental field and determines subsequent resources from both offspring quality and actual recruitment. It consequently shows more consistent advantages on LIRCMOP1–12. On LIRCMOP13–14, DPVAPS obtains better ranks. Its external archive and auxiliary-solution reinjection are more effective at preserving previously discovered high-quality search states on these two problems. These results also indicate that MFGCMOEA could further improve the retention of historical information under certain extreme constraint structures.

### 5.4. MW Test Suite

The IGD and HV results on the MW test suite are reported in Table 6. Compared with the preceding suites, MW contains more diverse constraint configurations and PF structures, making the relative strengths of different methods more problem-dependent. Nevertheless, MFGCMOEA achieves mean ranks of 1.79 for IGD and 1.71 for HV, ranks first on seven and eight problems, respectively, and retains a high proportion of significant advantages over methods including DRCMO, ANSGAIII, and AGEMOEAII. FLI has a mean rank of approximately 3.43 for both indicators and is one of the most consistent methods apart from MFGCMOEA. This shows the adaptability of landscape-based DE strategy selection to structurally different MW problems. Such regulation mainly determines how offspring are generated. It does not directly determine which auxiliary information should be retained or how evaluation resources should be divided between search processes. Beyond its search operators, MFGCMOEA forms resource evidence from developmental fronts, local propagation density, and actual recruitment. Computational effort can therefore follow effective contributions across stages, while the homeostatic basal growth term prevents short-term fluctuations from eliminating a search process’s recovery opportunities. RVEAa’s reference-vector mechanism performs particularly well on MW4 and MW14, TriP’s multirole cooperation is stronger on MW8 and MW13, and MW2 and MW6 demonstrate the effectiveness of FLI’s landscape-driven operator selection. These individual results show that reference vectors, three-population cooperation, and operator adaptation can each provide a useful bias for particular front shapes, constraint relationships, or local landscapes. MFGCMOEA is not best on every problem. However, its multi-morphogen field preserves several objective directions, developmental buffering adjusts infeasible-information use according to the current state, and dynamic allocation reduces redundancy from fixed dual-population budgets. These mechanisms lead to better overall robustness across the heterogeneous MW problems.

### 5.5. Overall Analysis

To further evaluate the computational cost of this coordination, Table 7 reports the mean runtime and standard deviation of the nine algorithms on the four benchmark suites. The runtime statistics are obtained under the same experimental setting used for the performance comparison. MFGCMOEA does not obtain the shortest runtime on every benchmark suite. It is the fastest method on CF, with an average runtime of 57.62, while ANSGAIII is faster on DASCMOP and MW and RVEAa is faster on LIRCMOP. Nevertheless, its computational cost remains comparable to the relatively lightweight single- or dual-process competitors. In particular, MFGCMOEA is substantially faster than DPVAPS, DRCMO, and TriP on all four suites. For example, on LIRCMOP, the mean runtimes of DPVAPS, DRCMO, and TriP are 1702.11, 1120.32, and 856.97, respectively, compared with 165.83 for MFGCMOEA. This indicates that the additional multi-morphogen propagation, developmental buffering, and homeostatic allocation do not translate into excessive wall-clock overhead in the current implementation. The runtime behavior is also consistent with the complexity analysis in Section 3.5. Although the multi-morphogen module introduces pairwise rank-space propagation and developmental-field calculations, these operations do not increase the asymptotic upper bound of the complete algorithm. The measured runtime further shows that the proposed mechanisms maintain a favorable computational-performance tradeoff rather than achieving better IGD and HV results through substantially greater execution time. In addition, several operations in MFGCMOEA, including pairwise distance evaluation, objective-channel signal propagation, and offspring evaluation, are naturally separable across candidates or channels and therefore provide opportunities for parallel implementation. Parallel speedup is not evaluated in the present study and remains a potential direction for reducing wall-clock cost on larger-scale problems.

**Figure 2:**
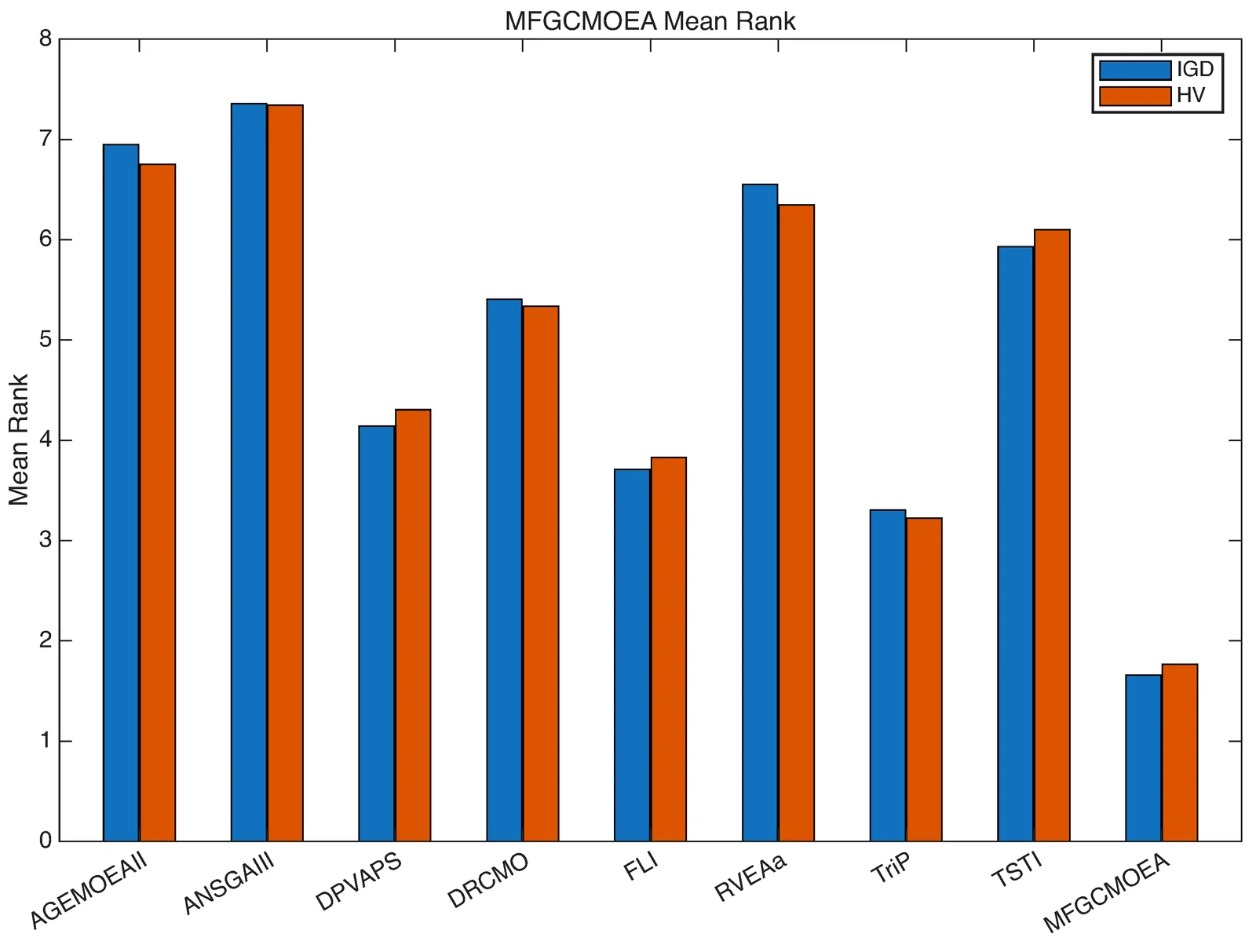
Mean IGD and HV ranks of the nine algorithms on 47 benchmark problems.

Across the four benchmark suites, Figure 2 shows that MFGCMOEA achieves overall mean ranks of approximately 1.66 for IGD and 1.74 for HV, both the lowest among the nine algorithms. TriP, FLI, and DPVAPS are the next most competitive methods in the aggregate ranking. AGEMOEAII, ANSGAIII, and RVEAa mainly emphasize objective-space geometry or reference directions, TSTI relies on stage-based pressure restructuring, DRCMO emphasizes dynamic constraint relaxation, and DPVAPS and TriP extend auxiliary search through additional populations or archives. The overall results indicate that strengthening only one aspect of constrained search is insufficient to consistently handle the different difficulties represented by CF, DASCMOP, LIRCMOP, and MW. MFGCMOEA instead coordinates objective-direction preservation, state-dependent developmental buffering, and resource regulation according to current search evidence, which provides a more consistent balance across heterogeneous constraint structures.

### 5.6. Convergence Analysis

**Figure 3:**
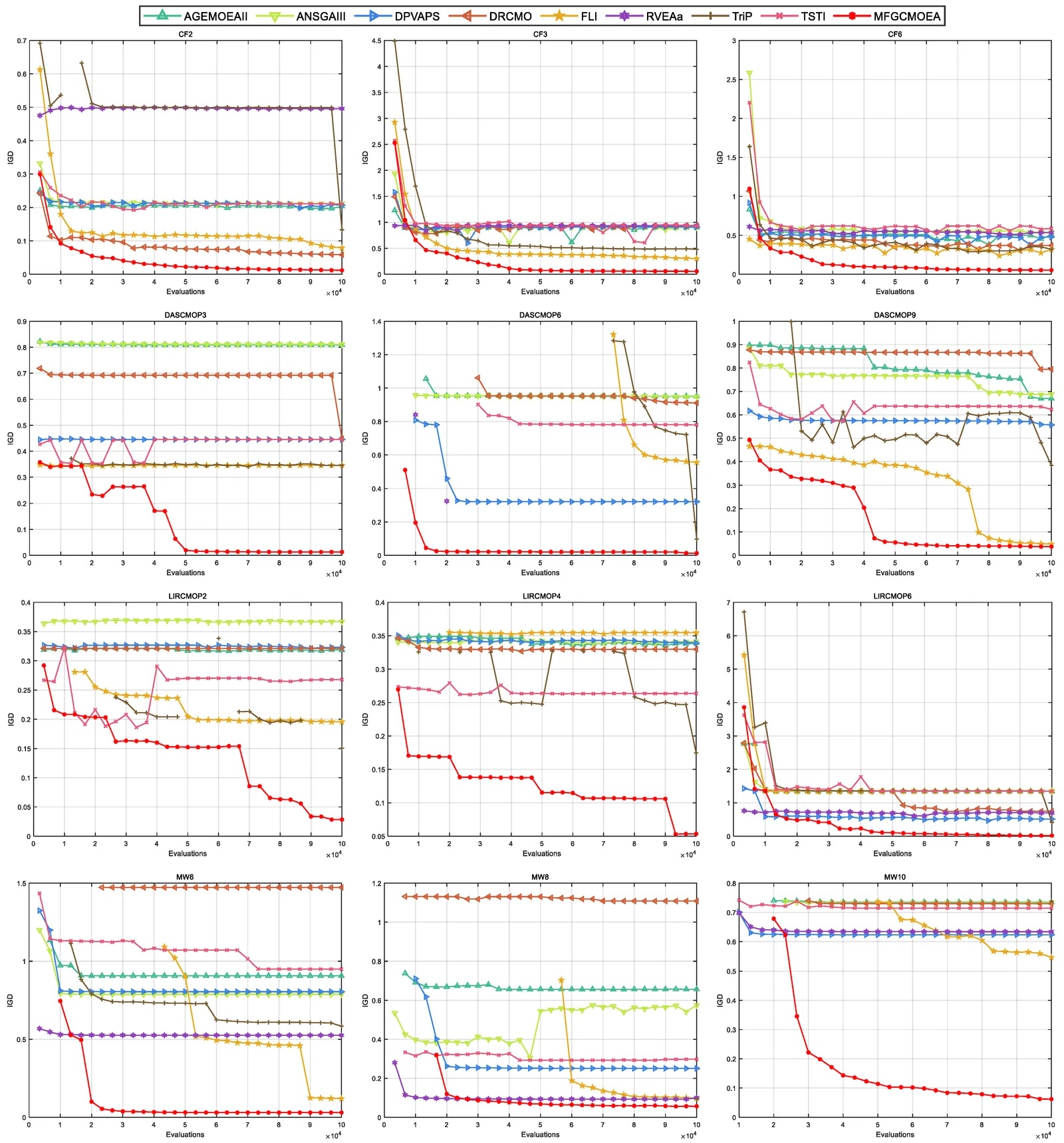
IGD convergence curves of the nine algorithms on representative test problems.

Figure 3 shows the IGD convergence trajectories of the nine algorithms on 12 representative problems. Overall, MFGCMOEA exhibits more sustained decreases under different constraint structures, rather than gaining its advantage solely from an early rapid decrease. On CF2, CF3, and CF6, front geometry estimation in AGEMOEAII, adaptive reference points in ANSGAIII, and reference-vector adjustment in RVEAa quickly establish objective-space distributions. Their curves, however, subsequently reach plateaus relatively early. This indicates that geometry or reference directions alone are insufficient to sustain progress across infeasible regions when constraints obstruct attainable directions. MFGCMOEA continues to decrease on all three problems, particularly reducing IGD during the middle and later stages on CF2 and CF3. The differences are more pronounced on DASCMOP3, DASCMOP6, and DASCMOP9. DPVAPS reduces the auxiliary population and gradually transfers resources to the main population, while TriP coordinates populations for the original, unconstrained, and relaxed problems. Both improve convergence during some stages, but their progress depends more on predefined roles or size changes. Although DRCMO’s dynamic relaxation facilitates crossing infeasible regions, it stagnates relatively early on these three problems. MFGCMOEA reaches low IGD values early on DASCMOP3 and DASCMOP6 and makes its decisive decrease earlier than FLI on DASCMOP9. FLI selects DE operators using fitness landscape indicators and still improves substantially late in the DASCMOP9 run, but responds later than MFGCMOEA. Operator changes alone therefore cannot fully replace coordinated regulation of auxiliary information and resources. On LIRCMOP2, LIRCMOP4, and LIRCMOP6, which contain large infeasible regions, TSTI restructures selection pressure using two stages and three indicators. DPVAPS, TriP, and DRCMO also sustain search through multiple populations or relaxation, but most curves exhibit extended middle-stage plateaus. MFGCMOEA makes repeated decreases on LIRCMOP2 and LIRCMOP4. On LIRCMOP6, it quickly leaves the high-IGD region and continues toward lower values. These trajectories indicate that morphogen channels and developmental buffering retain different objective directions while continually exploiting potential search routes within infeasible regions. MW5, MW8, and MW10 further illustrate robustness to complex constraint configurations. FLI improves substantially late in the MW5 run, RVEAa is competitive during the early and middle stages on MW8, and most competitors remain on relatively stable plateaus on MW10. MFGCMOEA achieves earlier or more sustained decreases on all three problems and ends with the lowest or near-lowest IGD. Overall, its advantage does not arise from a fixed stage or a single operator. It results from the combined roles of multi-morphogen fields in preserving objective tradeoffs, developmental buffers in sustaining infeasible-region search, and homeostatic allocation in supplying offspring to effective processes. These mechanisms yield more consistent convergence speed and late-stage improvement across different difficulty types.

### 5.7. Diversity Analysis

**Figure 4:**
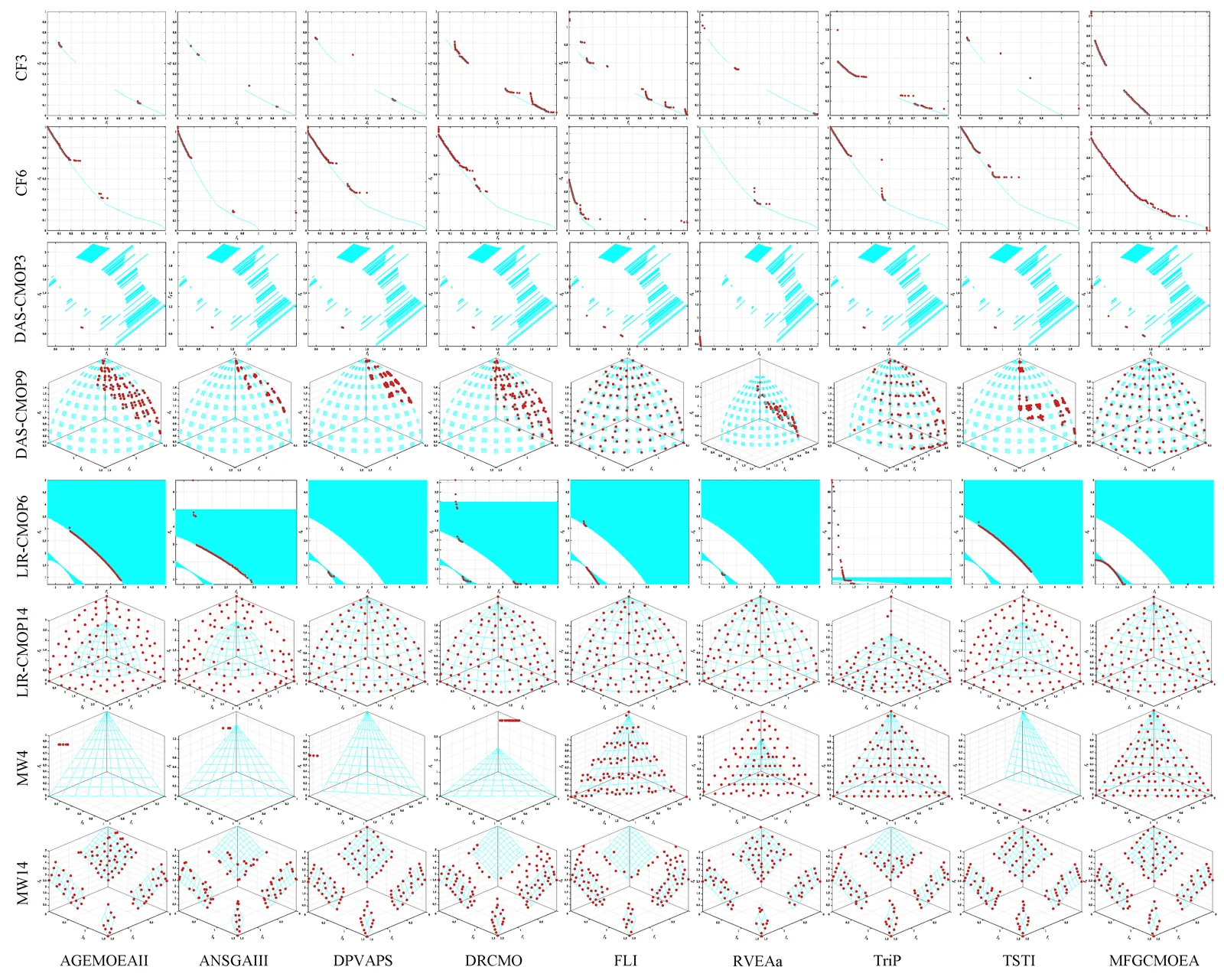
Population distributions of the nine algorithms on eight test problems.

Figure 4 compares the nine algorithms in terms of front coverage, local clustering, and agreement with the reference structure. On CF3, MFGCMOEA forms relatively continuous point sets in both disconnected segments. It also covers a substantial portion of the reference arc on CF6. In contrast, AGEMOEAII, which estimates front geometry [42], and ANSGAIII, which uses adaptive reference points [51], still exhibit local clustering and missing segments. Geometric dispersion therefore does not necessarily imply adequate coverage of constrained objective directions. On DASCMOP3, most solution sets concentrate at a few locations. Although MFGCMOEA retains solutions at more locations, substantial gaps remain, and the figure does not establish complete recovery of the reference structure. On DASCMOP9 and MW4, MFGCMOEA distributes solutions along the main surfaces. FLI, which switches differential operators using landscape indicators [46], and TriP, which coordinates the original, unconstrained, and relaxed problems [45], also provide broad coverage, demonstrating the effectiveness of operator adaptation and multirole search. DPVAPS supplements search information through auxiliary population size adjustment and an external archive [18], while DRCMO enhances exploration through dynamic relaxation and operator regulation [15]. Nevertheless, their displayed distributions remain biased toward local surfaces on DASCMOP9 and show clear clustering on MW4. Expanding the explored region and obtaining a balanced retained solution set are thus different tasks. On LIRCMOP6, MFGCMOEA provides relatively continuous coverage along the lower-left reference arc. TSTI’s two-stage, three-indicator selection [33] instead yields solutions mainly along the outer arc. Stronger late-stage feasibility pressure therefore does not automatically cover useful objective segments that have not yet been reached. On LIRCMOP14, MFGCMOEA, DPVAPS, DRCMO, and FLI all provide relatively complete surface coverage, with no clear difference in coverage extent. On MW14, MFGCMOEA retains solutions in all four disconnected patches, but local densities still differ. RVEAa, which adapts and regenerates reference vectors [43], also covers the patches well. The distributions alone therefore do not establish that MFGCMOEA is uniformly superior on these two structures. Overall, the advantage of MFGCMOEA lies in combining coverage of useful segments with reduced local clustering on several problems, rather than merely widening the point cloud. This observation agrees with its preservation of morphogen objective channels, use of developmental buffering to exploit promising infeasible directions, and restriction of contact inhibition to the critical developmental front. Homeostatic allocation also maintains opportunities for both search processes. These mechanistic interpretations are consistent with the figures, but the independent contributions of the mechanisms should still be assessed through ablation experiments.

### 5.8. Statistical Analysis

To assess the statistical significance of the performance differences, the problem-wise Wilcoxon signed-rank tests are first conducted for IGD and HV at a significance level of 0.05. The corresponding results are reported in Table 8 and Table 9. In addition, an across-problem Friedman test followed by Holm-corrected pairwise Wilcoxon tests is performed to account for multiple comparisons. Rank-biserial correlation, denoted by $r_{\mathrm{rb}}$, is further reported to quantify the magnitude and direction of the paired differences. A positive $r_{\mathrm{rb}}$ indicates an advantage of MFGCMOEA.

The across-problem analysis uses the 26 benchmark problems for which all nine algorithms have valid IGD and HV values. The Friedman test identifies significant differences among the algorithms for both IGD ($\chi^{2}=98.2974$, $p=9.51\times 10^{-18}$) and HV ($\chi^{2}=105.9060$, $p=2.64\times 10^{-19}$). On this common set, MFGCMOEA obtains the lowest mean rank of 1.7692 for both indicators. As shown in Table 10, the Holm-corrected pairwise tests remain significant at the 0.05 level for all eight competitors. Furthermore, $r_{\mathrm{rb}}$ ranges from 0.840 to 1.000 for IGD and from 0.835 to 1.000 for HV. According to the adopted criterion $|r_{\mathrm{rb}}|\geq 0.5$, all comparisons correspond to large effect sizes. These results indicate that the statistical differences are not explained solely by aggregate ranking, but are also supported by consistent paired performance differences.

The remaining 21 problems are excluded from the across-problem analysis because at least one algorithm contains an infeasible run and therefore has an undefined IGD or HV value under the adopted evaluation rule; no missing runs are involved in this exclusion. It is also worth noting that MFGCMOEA does not achieve feasibility on every benchmark, failing to obtain feasible solutions on CF8 and CF10. In contrast, it obtains feasible solutions in all 30 independent runs on every DASCMOP and LIRCMOP instance. The exclusions on these two suites mainly arise from infeasible runs of RVEA_*a*_. This complete-case treatment avoids replacing infeasible runs with artificial penalty values and separates feasibility discovery from approximation quality after feasible solutions are obtained.

### 5.9. Ablation and Mechanism Analysis

To evaluate the contribution of each component and further examine the internal behavior of MFGCMOEA, the original ablation study is extended with four mechanism-oriented variants. All variants use the same population size, total function-evaluation budget, and main-population environmental selection as the complete algorithm. Except for the mechanism explicitly modified in each variant, the remaining components are unchanged. Table 11 summarizes the complete set of variants.

#### 5.9.1. Ablation Results

Table 12 reports the overall mean ranks and rank-biserial effect sizes of MFGCMOEA and variants A–J. The mean rank is obtained by equally averaging the four suite-level mean ranks. For each variant, the rank-biserial correlation is calculated from finite paired problem means, with a positive value indicating better performance of MFGCMOEA. The number of valid paired problems is reported together with the effect size because some difficult instances do not provide finite performance values for all variants.

MFGCMOEA obtains the lowest overall mean ranks, 1.111 for IGD and 1.138 for HV. Variants B and C exhibit the largest rank deterioration among the individual component ablations. Removing developmental buffering increases the IGD and HV mean ranks to 8.773 and 8.157, whereas replacing the vector-valued developmental phenotype by a scalar produces ranks of 8.822 and 8.481. These results indicate that adaptive constraint tolerance and preservation of objective-specific developmental information are central to the auxiliary search. Variant A also degrades substantially, confirming that neighborhood propagation provides useful information beyond the intrinsic objective ranks. The deterioration of D further indicates that propagation-based contact inhibition contributes to distribution regulation on the critical developmental front. Variants E and F clarify the role of resource regulation. Fixing the offspring numbers throughout evolution results in mean ranks of 6.537 and 6.825, whereas removing only the homeostatic basal-growth term gives 6.198 and 6.633. Their rank-biserial correlations equal 1.000 for both indicators. Therefore, maintaining equal resources throughout evolution cannot fully exploit changes in the relative usefulness of the two search processes, while evidence-driven allocation without basal homeostatic protection is also less robust. These results are consistent with the intended role of the basal-growth term in limiting excessive responses to short-term contribution differences and preserving reproduction opportunities for a temporarily weaker search process. Variant G jointly removes propagation and buffering. Its mean ranks of 6.705 and 6.713 remain considerably worse than those of MFGCMOEA, but its degradation does not simply equal the accumulation of variants A and B. This result suggests that propagation and developmental buffering provide distinct contributions while interacting through the developmental-field construction. Variants H and I further examine the specific propagation and buffering formulations. Although their degradation is smaller than that of several complete component removals, both remain clearly inferior to MFGCMOEA, supporting the use of the soft propagation kernel and geometric developmental buffer rather than their sparse and arithmetic alternatives. Variant J uses the same DE/rand/1 operator in both offspring streams and yields mean ranks of 6.164 and 6.006. This confirms that operator heterogeneity contributes to the complete search process. However, variants A–I retain the original variation operators while modifying only selection, buffering, propagation, or allocation mechanisms and also exhibit substantial performance losses. Hence, the overall advantage cannot be attributed to the differential-evolution operators alone; it results from the interaction between the proposed developmental mechanisms and the complementary search processes.

**Figure 5:**
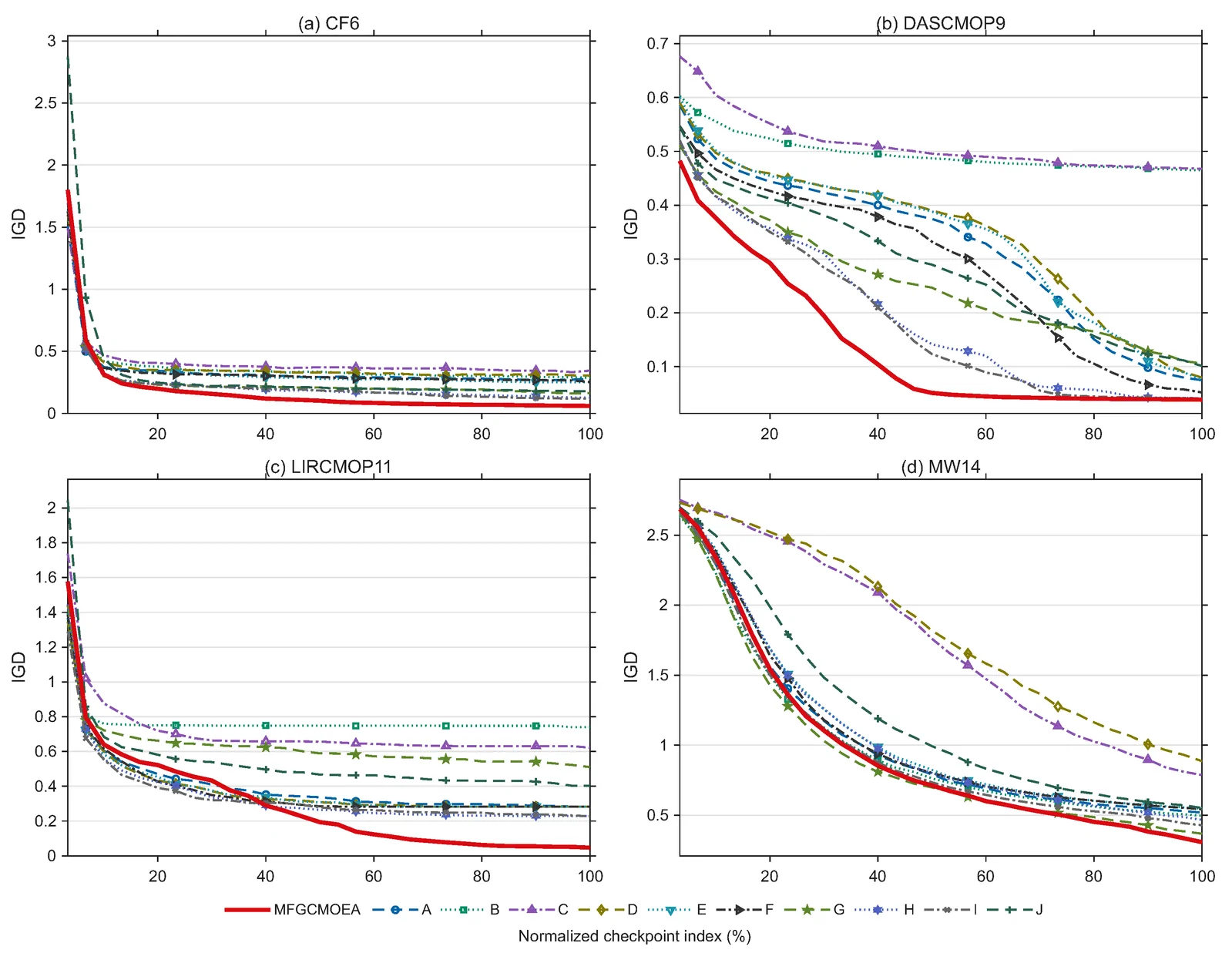
IGD convergence comparison between MFGCMOEA and variants A–J on four representative problems. The horizontal axis denotes the normalized archived checkpoint index.

**Figure 6:**
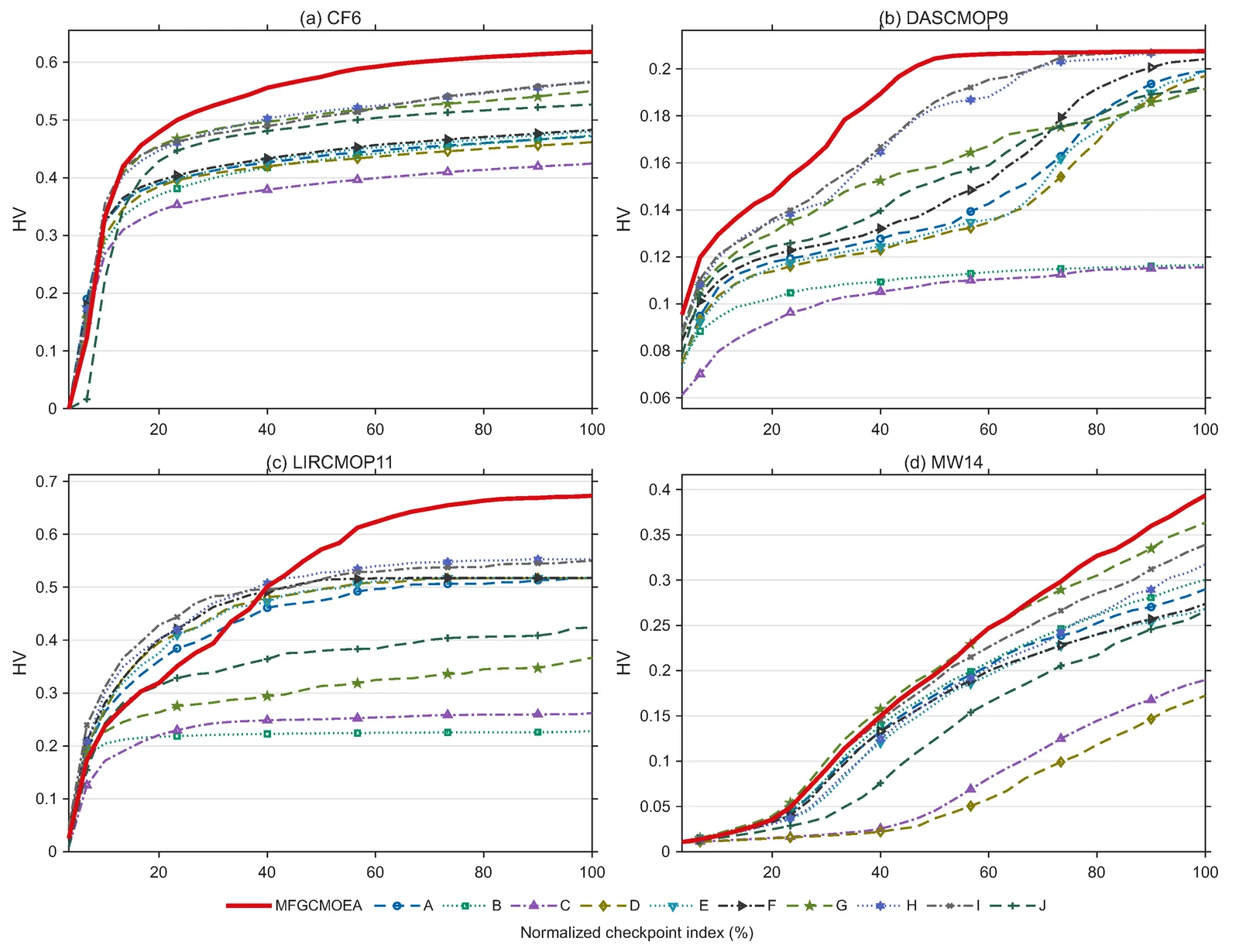
HV convergence comparison between MFGCMOEA and variants A–J on four representative problems.

Figure 5 and Figure 6 further compare the convergence behavior of MFGCMOEA and all ten variants on CF6, DASCMOP9, LIRCMOP11, and MW14. Each curve is averaged over 30 stored runs. Since the archived results retain ordered metric checkpoints but not their exact function-evaluation counters, the horizontal axis reports the normalized checkpoint index rather than absolute function evaluations. The terminal behavior of the curves is consistent with the overall statistical comparison, while the complete algorithm provides a common reference for observing how different component modifications alter the evolutionary trajectory.

**Figure 7:**
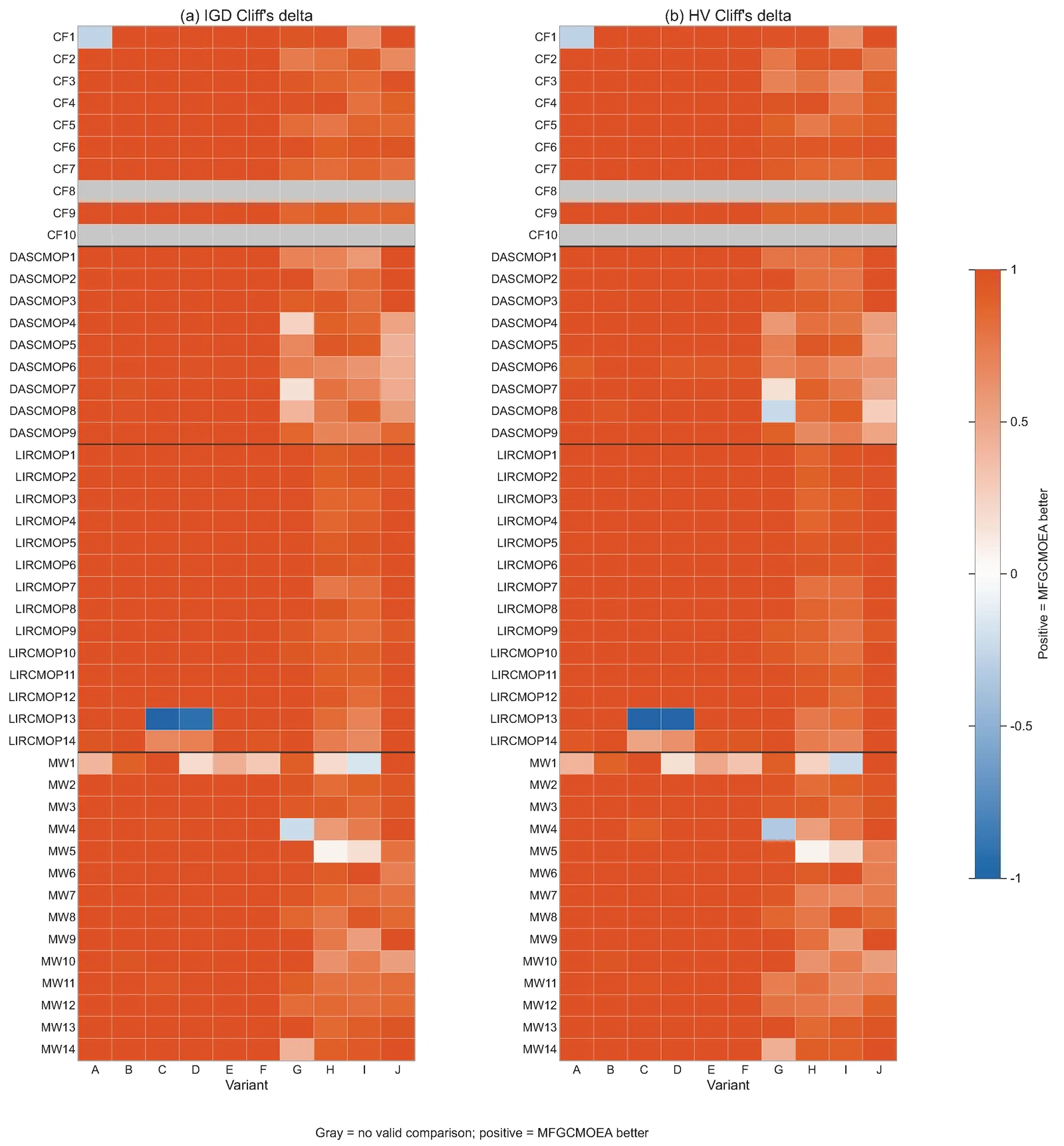
Per-problem Cliff’s δ between MFGCMOEA and variants A–J for IGD and HV. Positive values indicate better performance of MFGCMOEA, while gray cells denote unavailable effect sizes.

To complement the aggregate ranks with problem-level effect magnitudes, Figure 7 reports Cliff’s δ for MFGCMOEA against every variant on the 47 benchmark problems. Positive values consistently indicate an advantage for MFGCMOEA, with the direction reversed internally for IGD and HV according to their minimization and maximization preferences. CF8 and CF10 are left blank because the complete algorithm has no finite final IGD or HV values on these two problems. Among the 45 problems with finite final values for the complete algorithm, positive effect sizes dominate the comparison. Variants B, E, F, H, and J show positive Cliff’s δ on all 45 analyzable problems for both indicators. For B, all 45 effects are large for both IGD and HV. Variants A, C, D, and I contain only one negative comparison for each indicator, while G contains one negative IGD case and two negative HV cases. Large effects account for the majority of comparisons across all variants. The heatmap therefore supports the aggregate ranking results while also showing that the component contributions are not uniformly identical across all problem structures.

#### 5.9.2. Mechanism Diagnostics

In addition to the ablation variants, a diagnostic version of MFGCMOEA records intermediate search quantities without modifying any search, selection, or resource-allocation decision. The diagnostic experiment covers all 47 problems with 30 independent stored runs, yielding 705000 recorded generations. Table 13 first examines whether the resource evidence used by the allocation mechanism also predicts short-horizon improvement of the feasible approximation.

Here, ΔIGD=IGD(t)−IGD(t+1) and ΔHV=HV(t+1)−HV(t). None of the three evidence definitions exhibits a consistently positive monotonic relationship with short-horizon improvement. In particular, the proposed evidence has median correlations of −0.261 and −0.209 with subsequent IGD and HV improvement. Therefore, auxiliary recruitment should not be interpreted as a direct predictor of future performance. Its role is more appropriately understood as contemporaneous evidence describing how useful the generated offspring are to the current developmental search state. An offspring may preserve a useful auxiliary direction without immediately improving the feasible approximation. The strong degradation of variants E and F nevertheless indicates that using this current-state evidence within the proposed homeostatic allocation mechanism is beneficial to overall optimization performance.

Table 14 summarizes the main diagnostic quantities at the benchmark-suite level. Each entry is the median of problem-level summaries within the corresponding suite, so every problem contributes equally.

The rank-space representation remains highly stable across successive generations. The suite-level rank correlations range from 0.991 to 0.998, while the corresponding mean absolute rank differences remain between 0.019 and 0.034. The selected-set overlap ranges from 0.708 to 0.885 despite changes in candidate composition during evolution. These observations indicate that the candidate-set-relative representation adapts continuously rather than producing arbitrary changes in objective ordering.

The propagation statistics also clarify the behavior of the fully connected Cauchy kernel. Median normalized propagation weights are approximately $2.25\times 10^{-3}$–$2.30\times 10^{-3}$ across the four suites, while the effective neighborhood size remains between approximately 329 and 338 candidates. The developmental field therefore uses broad but graded information propagation rather than behaving as a narrowly truncated neighborhood graph. Combined with the deterioration of sparse-kernel variant H, this result indicates that broad soft propagation does not obscure the objective structure on the tested problems and that aggressive sparsification instead removes useful information.

The buffering statistics show a clear dependence on constraint structure. Median developmental buffers range from 0.820 to 0.911. The corresponding median relative constraint disturbances are approximately 0.006, 0.146, 0.246, and 0.283 for CF, DASCMOP, LIRCMOP, and MW, respectively, while the median residual stress is zero in all four suites. The selected infeasible rate ranges from 0.167 on CF to 0.917 on DASCMOP, confirming that the auxiliary population can retain substantial infeasible information when the constraint structure requires it. Moreover, mating events involving at least one retained infeasible auxiliary parent produce feasible offspring at nonzero conditional rates in every suite, ranging from 0.127 to 0.668. This observation does not establish a counterfactual causal contribution of the infeasible parent, but it confirms that retained infeasible individuals continue to participate in search processes that can lead to feasible offspring. Together with the deterioration of variant I, these results support the proposed geometric developmental buffer rather than an unconditional or purely arithmetic relaxation rule.

The root-mean-square constraint disturbance can be interpreted directly through Equation (9). For $k_{i}$ active constraints among *L* total constraints,

$$c_{i}=\sqrt{\frac{k_{i}}{L}}\sqrt{\frac{1}{k_{i}}\sum_{l\in A_{i}}{\left(r_{il}^{c}\right)}^{2}}.$$

This form simultaneously reflects violation breadth and relative severity. For example, when L=4, one violation with relative rank 0.8 gives $c_{i}=0.4$, two violations with ranks 0.4 give $c_{i}\approx 0.283$, and four violations with ranks 0.4 again give $c_{i}=0.4$. Conversely, maintaining an active-constraint proportion of 1/4 with relative rank 0.4 gives $c_{i}=0.2$ for $\left(L,k_{i}\right)=\left(4,1\right)$, (8,2), and (16,4). Hence, the disturbance does not depend on the absolute number of constraints alone; it combines the proportion of active constraints with their normalized severity.

Finally, the single-front special case of the resource-quality model is examined over all recorded generations. The condition $\nu_{\max}=1$ is not observed in any of the 705,000 diagnostic generations, corresponding to an empirical frequency of zero. Therefore, although $q_{i}=e_{i}=1$ theoretically makes density adjustment inactive when all candidates belong to one developmental front, this degenerate case does not affect the reported benchmark experiments. If it occurs, the resulting equal quality evidence naturally returns the allocation toward a neutral balance rather than introducing an artificial density preference.

Overall, the ablation and diagnostic results provide complementary evidence for the proposed design. The A–J comparisons show that objective-channel propagation, developmental buffering, vector-valued phenotypes, critical-front contact inhibition, dynamic resource allocation, homeostatic basal growth, propagation topology, buffer formulation, and complementary variation operators all affect final performance. The diagnostic results further show that the rank-space representation remains stable, that morphogen propagation operates over a broad but graded neighborhood, and that adaptive buffering preserves infeasible search information without permanently removing constraint pressure.

## 6. Conclusions

This paper proposed MFGCMOEA, a dual-population constrained multiobjective evolutionary algorithm driven by multi-morphogen developmental fields. The main population maintains strict feasibility-oriented convergence, whereas the auxiliary population represents each objective through an independent morphogen channel and propagates developmental signals in objective-rank space. The propagated information is used to construct individual constraint buffers and multidimensional developmental phenotypes, allowing promising infeasible search directions to be retained without reducing the objective information to a single scalar criterion. Critical-front contact inhibition further regulates the distribution among candidates at the same developmental level. A homeostatic resource-allocation mechanism reuses developmental quality and offspring recruitment information to adjust the offspring budget between the two search processes, while the symmetric basal-growth term limits excessive allocation polarization. Experiments on 47 benchmark problems from four test suites show that MFGCMOEA achieves the lowest overall IGD and HV mean ranks among the compared algorithms and maintains competitive runtime. The statistical, convergence, distribution, ablation, and mechanism-diagnostic results further support the contributions of multi-channel information preservation, developmental buffering, propagation, and homeostatic resource regulation.

Several limitations remain. First, the rank-space representation is relative to the current candidate set, and although the diagnostic results show high stability across successive generations, its behavior may become more sensitive when population composition changes sharply in larger or higher-dimensional problems. Second, the fully connected propagation mechanism requires pairwise relationships among candidates and therefore introduces additional practical computational cost even though it does not increase the asymptotic upper bound of the current implementation; more efficient sparse or parallel implementations deserve further investigation. Third, the recruitment signal used for resource allocation reflects the current developmental search state rather than serving as a direct short-horizon predictor of subsequent IGD or HV improvement. In addition, coverage gaps and local density differences remain on some complex or disconnected constrained Pareto fronts, and the present validation is limited to benchmark problems. Future work will therefore investigate more efficient propagation and parallel implementation, improved retention of useful historical search states, and adaptive distribution regulation for undercovered regions. The algorithm will also be evaluated on real engineering problems with different constraint structures, objective dimensions, and evaluation budgets to further examine the applicability and limitations of developmental buffering and dynamic resource allocation.

## Figures and Tables

**Table 1:** Feature-level comparison between MFGCMOEA and closely related dual-population CMOEAs.

Method	Auxiliary-Search Representation	Constraint Treatment	Resource Regulation	Main Distinction
DPVAPS [18]	Unconstrained auxiliary population	Auxiliary search without direct constraint pressure	Adaptive auxiliary- population size	Resource adjustment is realized mainly through population-size adaptation
DRCMO [15]	Cooperative main and auxiliary populations	Dynamic constraint relaxation	Resource use follows the dual-population search process	Adaptation mainly targets the constraint-relaxation state of auxiliary search
MGCMOEA [19]	Cooperative main and auxiliary populations	Constraint-aware cooperative search	Multicriteria game-based offspring allocation	Resource allocation is driven by game-based performance evaluation
MFGCMOEA	Objective-wise vector developmental phenotype	Morphogen-dependent individual constraint buffering	Developmental quality, recruitment evidence, and homeostatic basal growth	Rank-space propagation couples objective representation, constraint tolerance, and resource evidence

**Table 2:** Main parameter settings of the compared algorithms.

Algorithm	Parameter Settings
AGEMOEAII	$p_{c}=1$, $p_{m}=1/D$, $\eta_{c}=20$, $\eta_{m}=20$
ANSGAIII	$p_{c}=1$, $p_{m}=1/D$, $\eta_{c}=20$, $\eta_{m}=20$
DPVAPS	$p_{c}=1$, $p_{m}=1/D$, $\eta_{c}=20$, $\eta_{m}=20$
DRCMO	–
FLI	–
RVEA_*a*_	α=2, $f_{r}=0.1$, $p_{c}=1$, $p_{m}=1/D$, $\eta_{c}=20$, $\eta_{m}=20$
TriP	δ=0.9, $n_{r}=2$, F=0.5, CR=1, $p_{m}=1/D$, $\eta_{m}=20$
TSTI	$\epsilon_{0}=0.05$, ρ=1.01, ${\mathrm{FE}}_{s}=0.4\;\mathrm{maxFE}$, $p_{c}=1$, $p_{m}=1/D$, $\eta_{c}=20$, $\eta_{m}=20$
MFGCMOEA	p=0.1, F={0.6,0.8,1.0}, $C_{R}=\left\{0.1,0.2,1.0\right\}$, $p_{m}=1/D$, $\eta_{m}=20$, $\lambda_{h}=1$

**Table 3:** IGD and HV results on the CF test suite.

Problem	Metric	AGEMOEAII	ANSGAIII	DPVAPS	DRCMO	FLI	RVEAa	TriP	TSTI	MFGCMOEA
CF1	IGD HV	$4.37\times 10^{-2}$ ($3.38\times 10^{-3}$)+ $5.12\times 10^{-1}$ ($4.12\times 10^{-3}$)+	$4.35\times 10^{-2}$ ($2.38\times 10^{-3}$)+ $5.12\times 10^{-1}$ ($3.07\times 10^{-3}$)+	$1.93\times 10^{-2}$ ($7.39\times 10^{-4}$)+ $5.41\times 10^{-1}$ ($9.24\times 10^{-4}$)+	$1.19\times 10^{-2}$ ($2.53\times 10^{-3}$)+ $5.52\times 10^{-1}$ ($3.07\times 10^{-3}$)+	$4.45\times 10^{-3}$ **($2.80\times 10^{-4}$)−** $5.61\times 10^{-1}$ **($3.43\times 10^{-4}$)−**	$7.68\times 10^{-2}$ ($1.70\times 10^{-2}$)+ $4.82\times 10^{-1}$ ($1.91\times 10^{-2}$)+	$4.63\times 10^{-3}$ ($8.92\times 10^{-4}$)− $5.60\times 10^{-1}$ ($1.14\times 10^{-3}$)−	$5.03\times 10^{-2}$ ($2.82\times 10^{-3}$)+ $5.04\times 10^{-1}$ ($3.32\times 10^{-3}$)+	$5.36\times 10^{-3}$ ($3.05\times 10^{-4}$) $5.60\times 10^{-1}$ ($3.70\times 10^{-4}$)
CF2	IGD HV	$1.54\times 10^{-1}$ ($2.88\times 10^{-2}$)+ $4.90\times 10^{-1}$ ($2.11\times 10^{-2}$)+	$1.45\times 10^{-1}$ ($2.93\times 10^{-2}$)+ $5.13\times 10^{-1}$ ($2.39\times 10^{-2}$)+	$1.44\times 10^{-1}$ ($2.82\times 10^{-2}$)+ $5.11\times 10^{-1}$ ($2.39\times 10^{-2}$)+	$4.41\times 10^{-2}$ ($1.03\times 10^{-2}$)+ $6.12\times 10^{-1}$ ($1.93\times 10^{-2}$)+	$6.26\times 10^{-2}$ ($6.58\times 10^{-3}$)+ $5.79\times 10^{-1}$ ($8.88\times 10^{-3}$)+	$1.24\times 10^{-1}$ ($7.70\times 10^{-2}$)+ $5.26\times 10^{-1}$ ($4.84\times 10^{-2}$)+	$6.57\times 10^{-2}$ ($1.79\times 10^{-2}$)+ $5.95\times 10^{-1}$ ($1.65\times 10^{-2}$)+	$1.52\times 10^{-1}$ ($2.32\times 10^{-2}$)+ $4.97\times 10^{-1}$ ($2.40\times 10^{-2}$)+	$1.43\times 10^{-2}$ **($1.41\times 10^{-3}$)** $6.60\times 10^{-1}$ **($1.54\times 10^{-3}$)**
CF3	IGD HV	$5.40\times 10^{-1}$ ($1.30\times 10^{-1}$)+ $1.04\times 10^{-1}$ ($3.54\times 10^{-2}$)+	$4.41\times 10^{-1}$ ($1.66\times 10^{-1}$)+ $1.38\times 10^{-1}$ ($4.28\times 10^{-2}$)+	$4.98\times 10^{-1}$ ($1.59\times 10^{-1}$)+ $1.19\times 10^{-1}$ ($3.98\times 10^{-2}$)+	$4.66\times 10^{-1}$ ($2.72\times 10^{-1}$)+ $1.33\times 10^{-1}$ ($8.04\times 10^{-2}$)+	$2.29\times 10^{-1}$ ($3.32\times 10^{-2}$)+ $1.80\times 10^{-1}$ ($2.98\times 10^{-2}$)+	$7.68\times 10^{-1}$ ($1.33\times 10^{-1}$)+ $3.73\times 10^{-2}$ ($2.81\times 10^{-2}$)+	$2.95\times 10^{-1}$ ($7.48\times 10^{-2}$)+ $1.88\times 10^{-1}$ ($3.20\times 10^{-2}$)+	$5.19\times 10^{-1}$ ($1.72\times 10^{-1}$)+ $1.10\times 10^{-1}$ ($3.41\times 10^{-2}$)+	$7.30\times 10^{-2}$ **($6.77\times 10^{-3}$)** $2.77\times 10^{-1}$ **($1.70\times 10^{-2}$)**
CF4	IGD HV	$5.11\times 10^{-1}$ ($6.61\times 10^{-2}$)+ $1.72\times 10^{-1}$ ($4.17\times 10^{-2}$)+	$4.12\times 10^{-1}$ ($7.49\times 10^{-2}$)+ $2.13\times 10^{-1}$ ($5.05\times 10^{-2}$)+	$4.37\times 10^{-1}$ ($6.49\times 10^{-2}$)+ $1.99\times 10^{-1}$ ($4.20\times 10^{-2}$)+	$3.75\times 10^{-1}$ ($1.84\times 10^{-1}$)+ $2.18\times 10^{-1}$ ($1.24\times 10^{-1}$)+	$1.12\times 10^{-1}$ ($6.07\times 10^{-3}$)+ $3.98\times 10^{-1}$ ($3.03\times 10^{-3}$)+	$4.95\times 10^{-1}$ ($7.80\times 10^{-2}$)+ $1.93\times 10^{-1}$ ($4.66\times 10^{-2}$)+	$2.41\times 10^{-1}$ ($4.77\times 10^{-2}$)+ $3.04\times 10^{-1}$ ($2.35\times 10^{-2}$)+	$4.67\times 10^{-1}$ ($8.28\times 10^{-2}$)+ $1.83\times 10^{-1}$ ($4.60\times 10^{-2}$)+	$6.53\times 10^{-2}$ **($8.38\times 10^{-3}$)** $4.42\times 10^{-1}$ **($1.22\times 10^{-2}$)**
CF5	IGD HV	$6.21\times 10^{-1}$ ($3.36\times 10^{-2}$)+ $1.16\times 10^{-1}$ ($2.69\times 10^{-2}$)+	$6.38\times 10^{-1}$ ($2.78\times 10^{-2}$)+ $1.08\times 10^{-1}$ ($2.46\times 10^{-2}$)+	$6.13\times 10^{-1}$ ($3.69\times 10^{-2}$)+ $1.24\times 10^{-1}$ ($2.20\times 10^{-2}$)+	$4.27\times 10^{-1}$ ($1.31\times 10^{-1}$)+ $1.63\times 10^{-1}$ ($9.05\times 10^{-2}$)+	$1.13\times 10^{0}$ ($4.39\times 10^{-1}$)+ $1.90\times 10^{-4}$ ($1.04\times 10^{-3}$)+	$6.24\times 10^{-1}$ ($1.76\times 10^{-1}$)+ $1.28\times 10^{-1}$ ($4.09\times 10^{-2}$)+	$3.98\times 10^{-1}$ ($5.17\times 10^{-2}$)+ $2.32\times 10^{-1}$ ($3.80\times 10^{-2}$)+	$6.15\times 10^{-1}$ ($4.10\times 10^{-2}$)+ $1.23\times 10^{-1}$ ($2.28\times 10^{-2}$)+	$2.05\times 10^{-1}$ **($3.81\times 10^{-2}$)** $3.13\times 10^{-1}$ **($2.52\times 10^{-2}$)**
CF6	IGD HV	$4.62\times 10^{-1}$ ($1.96\times 10^{-2}$)+ $3.61\times 10^{-1}$ ($1.33\times 10^{-2}$)+	$4.28\times 10^{-1}$ ($4.40\times 10^{-2}$)+ $3.59\times 10^{-1}$ ($4.80\times 10^{-2}$)+	$4.31\times 10^{-1}$ ($2.28\times 10^{-2}$)+ $3.84\times 10^{-1}$ ($1.72\times 10^{-2}$)+	$2.71\times 10^{-1}$ ($8.78\times 10^{-2}$)+ $4.93\times 10^{-1}$ ($5.51\times 10^{-2}$)+	$2.37\times 10^{-1}$ ($3.73\times 10^{-2}$)+ $4.82\times 10^{-1}$ ($1.63\times 10^{-2}$)+	$4.59\times 10^{-1}$ ($4.15\times 10^{-2}$)+ $3.51\times 10^{-1}$ ($3.54\times 10^{-2}$)+	$2.61\times 10^{-1}$ ($2.81\times 10^{-2}$)+ $4.84\times 10^{-1}$ ($2.13\times 10^{-2}$)+	$4.66\times 10^{-1}$ ($4.17\times 10^{-2}$)+ $3.38\times 10^{-1}$ ($8.37\times 10^{-2}$)+	$6.05\times 10^{-2}$ **($3.64\times 10^{-3}$)** $6.18\times 10^{-1}$ **($3.08\times 10^{-3}$)**
CF7	IGD HV	$6.07\times 10^{-1}$ ($1.03\times 10^{-1}$)+ $2.05\times 10^{-1}$ ($8.49\times 10^{-2}$)+	$5.85\times 10^{-1}$ ($8.43\times 10^{-2}$)+ $2.12\times 10^{-1}$ ($7.91\times 10^{-2}$)+	$6.00\times 10^{-1}$ ($1.01\times 10^{-1}$)+ $1.92\times 10^{-1}$ ($8.88\times 10^{-2}$)+	$5.24\times 10^{-1}$ ($1.70\times 10^{-1}$)+ $2.16\times 10^{-1}$ ($1.16\times 10^{-1}$)+	$2.55\times 10^{0}$ ($6.99\times 10^{-1}$)+ $0.00\times 10^{0}$ ($0.00\times 10^{0}$)+	$6.77\times 10^{-1}$ ($7.97\times 10^{-2}$)+ $2.00\times 10^{-1}$ ($6.19\times 10^{-2}$)+	$4.23\times 10^{-1}$ ($9.96\times 10^{-2}$)+ $2.52\times 10^{-1}$ ($8.90\times 10^{-2}$)+	$6.16\times 10^{-1}$ ($7.00\times 10^{-2}$)+ $1.98\times 10^{-1}$ ($7.24\times 10^{-2}$)+	$1.58\times 10^{-1}$ **($3.10\times 10^{-2}$)** $5.04\times 10^{-1}$ **($3.20\times 10^{-2}$)**
CF8	IGD HV	NaN (0.00%)= NaN (0.00%)=	NaN (0.00%)= NaN (0.00%)=	$4.81\times 10^{-1}$ ($6.83\times 10^{-2}$)− $9.84\times 10^{-2}$ ($1.43\times 10^{-2}$)−	NaN (43.87%)− NaN (43.87%)−	$3.85\times 10^{-1}$ ($1.88\times 10^{-2}$)− $1.72\times 10^{-1}$ ($9.07\times 10^{-3}$)−	$2.55\times 10^{-1}$ **($2.14\times 10^{-2}$)−** $3.01\times 10^{-1}$ **($1.06\times 10^{-2}$)−**	$3.35\times 10^{-1}$ ($2.92\times 10^{-2}$)− $1.59\times 10^{-1}$ ($2.25\times 10^{-2}$)−	NaN (26.67%)− NaN (26.67%)−	NaN (0.00%) NaN (0.00%)
CF9	IGD HV	$9.45\times 10^{-1}$ ($2.39\times 10^{-2}$)+ $8.79\times 10^{-2}$ ($1.66\times 10^{-2}$)+	$9.41\times 10^{-1}$ ($4.04\times 10^{-4}$)+ $9.09\times 10^{-2}$ ($6.23\times 10^{-7}$)+	$2.13\times 10^{-1}$ ($5.51\times 10^{-3}$)+ $2.48\times 10^{-1}$ ($6.70\times 10^{-3}$)+	$6.79\times 10^{-1}$ ($3.69\times 10^{-1}$)+ $1.57\times 10^{-1}$ ($9.88\times 10^{-2}$)+	$1.66\times 10^{-1}$ ($6.18\times 10^{-3}$)+ $2.95\times 10^{-1}$ ($5.13\times 10^{-3}$)+	$1.36\times 10^{-1}$ **($5.86\times 10^{-3}$)−** $3.32\times 10^{-1}$ **($2.70\times 10^{-3}$)−**	$1.95\times 10^{-1}$ ($9.55\times 10^{-3}$)+ $2.60\times 10^{-1}$ ($1.02\times 10^{-2}$)+	$9.21\times 10^{-1}$ ($6.73\times 10^{-2}$)+ $9.09\times 10^{-2}$ ($1.03\times 10^{-4}$)+	$1.47\times 10^{-1}$ ($7.63\times 10^{-3}$) $3.23\times 10^{-1}$ ($1.03\times 10^{-2}$)
CF10	IGD HV	NaN (0.00%)= NaN (0.00%)=	NaN (0.00%)= NaN (0.00%)=	$4.54\times 10^{-1}$ **($7.59\times 10^{-2}$)−** $8.64\times 10^{-2}$ ($2.92\times 10^{-3}$)−	NaN (0.00%)= NaN (0.00%)=	$2.90\times 10^{0}$ ($2.03\times 10^{0}$)− $0.00\times 10^{0}$ ($0.00\times 10^{0}$)−	$4.74\times 10^{-1}$ ($9.46\times 10^{-2}$)− $1.07\times 10^{-1}$ **($3.81\times 10^{-2}$)−**	$8.20\times 10^{-1}$ ($6.54\times 10^{-1}$)− $3.42\times 10^{-2}$ ($3.38\times 10^{-2}$)−	NaN (0.00%)= NaN (0.00%)=	NaN (0.00%) NaN (0.00%)
+/−/=	IGD HV	8/0/2 8/0/2	8/0/2 8/0/2	8/2/0 8/2/0	8/1/1 8/1/1	7/3/0 7/3/0	7/3/0 7/3/0	7/3/0 7/3/0	8/1/1 8/1/1	– –

**Table 4:** IGD and HV results on the DASCMOP test suite.

Problem	Metric	AGEMOEAII	ANSGAIII	DPVAPS	DRCMO	FLI	RVEAa	TriP	TSTI	MFGCMOEA
DASCMOP1	IGD HV	$8.05\times 10^{-1}$ ($1.08\times 10^{-2}$)+ $0.00\times 10^{0}$ ($0.00\times 10^{0}$)+	$8.04\times 10^{-1}$ ($8.95\times 10^{-3}$)+ $0.00\times 10^{0}$ ($0.00\times 10^{0}$)+	$7.66\times 10^{-1}$ ($8.62\times 10^{-3}$)+ $3.08\times 10^{-4}$ ($4.85\times 10^{-4}$)+	$7.27\times 10^{-1}$ ($1.14\times 10^{-1}$)+ $1.00\times 10^{-2}$ ($3.12\times 10^{-2}$)+	$4.90\times 10^{-3}$ ($4.65\times 10^{-4}$)+ $2.10\times 10^{-1}$ ($4.40\times 10^{-4}$)+	NaN (0.00%)+ NaN (0.00%)+	$6.60\times 10^{-1}$ ($3.74\times 10^{-2}$)+ $2.35\times 10^{-2}$ ($1.51\times 10^{-2}$)+	$7.19\times 10^{-1}$ ($1.23\times 10^{-2}$)+ $8.81\times 10^{-3}$ ($2.48\times 10^{-3}$)+	$3.58\times 10^{-3}$ **($6.94\times 10^{-4}$)** $2.12\times 10^{-1}$ **($5.26\times 10^{-4}$)**
DASCMOP2	IGD HV	$3.17\times 10^{-1}$ ($2.73\times 10^{-2}$)+ $2.41\times 10^{-1}$ ($2.65\times 10^{-3}$)+	$3.04\times 10^{-1}$ ($2.68\times 10^{-2}$)+ $2.40\times 10^{-1}$ ($2.41\times 10^{-3}$)+	$2.53\times 10^{-1}$ ($1.83\times 10^{-2}$)+ $2.56\times 10^{-1}$ ($2.05\times 10^{-3}$)+	$2.54\times 10^{-1}$ ($4.91\times 10^{-2}$)+ $2.56\times 10^{-1}$ ($1.46\times 10^{-2}$)+	$5.23\times 10^{-3}$ ($1.05\times 10^{-4}$)+ $3.54\times 10^{-1}$ ($9.85\times 10^{-5}$)+	NaN (0.00%)+ NaN (0.00%)+	$1.72\times 10^{-1}$ ($2.61\times 10^{-2}$)+ $2.85\times 10^{-1}$ ($1.02\times 10^{-2}$)+	$3.16\times 10^{-1}$ ($2.29\times 10^{-2}$)+ $2.57\times 10^{-1}$ ($2.48\times 10^{-3}$)+	$4.01\times 10^{-3}$ **($7.30\times 10^{-5}$)** $3.56\times 10^{-1}$ **($6.66\times 10^{-5}$)**
DASCMOP3	IGD HV	$3.88\times 10^{-1}$ ($1.18\times 10^{-1}$)+ $1.89\times 10^{-1}$ ($5.73\times 10^{-2}$)+	$4.22\times 10^{-1}$ ($1.16\times 10^{-1}$)+ $1.86\times 10^{-1}$ ($5.86\times 10^{-2}$)+	$3.56\times 10^{-1}$ ($2.44\times 10^{-2}$)+ $2.07\times 10^{-1}$ ($3.42\times 10^{-4}$)+	$3.99\times 10^{-1}$ ($5.05\times 10^{-2}$)+ $2.05\times 10^{-1}$ ($1.74\times 10^{-2}$)+	$3.44\times 10^{-1}$ ($3.63\times 10^{-4}$)+ $2.09\times 10^{-1}$ ($1.10\times 10^{-4}$)+	NaN (0.00%)+ NaN (0.00%)+	$3.45\times 10^{-1}$ ($3.87\times 10^{-4}$)+ $2.08\times 10^{-1}$ ($1.15\times 10^{-4}$)+	$3.67\times 10^{-1}$ ($4.00\times 10^{-2}$)+ $2.08\times 10^{-1}$ ($4.05\times 10^{-4}$)+	$4.31\times 10^{-2}$ **($6.11\times 10^{-2}$)** $3.04\times 10^{-1}$ **($1.94\times 10^{-2}$)**
DASCMOP4	IGD HV	$3.70\times 10^{-1}$ ($2.74\times 10^{-2}$)+ $5.98\times 10^{-2}$ ($5.93\times 10^{-3}$)+	$5.44\times 10^{-1}$ ($4.77\times 10^{-2}$)+ $3.11\times 10^{-2}$ ($7.40\times 10^{-3}$)+	$3.23\times 10^{-3}$ ($1.33\times 10^{-3}$)+ $1.98\times 10^{-1}$ ($3.71\times 10^{-3}$)+	$2.25\times 10^{-1}$ ($1.52\times 10^{-1}$)+ $1.13\times 10^{-1}$ ($6.07\times 10^{-2}$)+	$2.17\times 10^{-1}$ ($1.64\times 10^{-1}$)+ $1.19\times 10^{-1}$ ($6.08\times 10^{-2}$)+	NaN (0.00%)+ NaN (0.00%)+	$1.30\times 10^{-2}$ ($7.46\times 10^{-3}$)+ $1.92\times 10^{-1}$ ($1.62\times 10^{-3}$)+	$3.09\times 10^{-1}$ ($5.14\times 10^{-2}$)+ $8.03\times 10^{-2}$ ($1.50\times 10^{-2}$)+	$2.12\times 10^{-3}$ **($5.24\times 10^{-4}$)** $2.03\times 10^{-1}$ **($3.53\times 10^{-4}$)**
DASCMOP5	IGD HV	$5.77\times 10^{-1}$ ($4.12\times 10^{-2}$)+ $5.59\times 10^{-2}$ ($6.85\times 10^{-3}$)+	$6.90\times 10^{-1}$ ($4.70\times 10^{-2}$)+ $3.82\times 10^{-2}$ ($8.80\times 10^{-3}$)+	$3.04\times 10^{-3}$ **($1.89\times 10^{-4}$)−** $3.51\times 10^{-1}$ **($9.71\times 10^{-5}$)−**	$3.28\times 10^{-1}$ ($2.41\times 10^{-1}$)+ $1.71\times 10^{-1}$ ($1.29\times 10^{-1}$)+	$1.46\times 10^{-1}$ ($1.91\times 10^{-1}$)+ $2.65\times 10^{-1}$ ($1.10\times 10^{-1}$)+	NaN (0.00%)+ NaN (0.00%)+	$5.16\times 10^{-3}$ ($4.98\times 10^{-4}$)+ $3.49\times 10^{-1}$ ($4.16\times 10^{-4}$)+	$2.54\times 10^{-1}$ ($1.54\times 10^{-1}$)+ $1.95\times 10^{-1}$ ($9.06\times 10^{-2}$)+	$3.14\times 10^{-3}$ ($1.51\times 10^{-4}$) $3.51\times 10^{-1}$ ($1.37\times 10^{-4}$)
DASCMOP6	IGD HV	$7.00\times 10^{-1}$ ($7.54\times 10^{-2}$)+ $2.51\times 10^{-2}$ ($1.03\times 10^{-2}$)+	$7.08\times 10^{-1}$ ($6.02\times 10^{-2}$)+ $2.17\times 10^{-2}$ ($9.24\times 10^{-3}$)+	$1.01\times 10^{-1}$ ($6.46\times 10^{-2}$)+ $2.77\times 10^{-1}$ ($1.67\times 10^{-2}$)+	$3.75\times 10^{-1}$ ($2.51\times 10^{-1}$)+ $1.34\times 10^{-1}$ ($1.18\times 10^{-1}$)+	$3.26\times 10^{-1}$ ($1.74\times 10^{-1}$)+ $1.47\times 10^{-1}$ ($9.38\times 10^{-2}$)+	NaN (0.00%)+ NaN (0.00%)+	$5.75\times 10^{-2}$ ($2.36\times 10^{-2}$)+ $2.95\times 10^{-1}$ ($5.60\times 10^{-3}$)+	$6.22\times 10^{-1}$ ($5.74\times 10^{-2}$)+ $3.57\times 10^{-2}$ ($9.73\times 10^{-3}$)+	$1.81\times 10^{-2}$ **($3.03\times 10^{-3}$)** $3.12\times 10^{-1}$ **($5.69\times 10^{-4}$)**
DASCMOP7	IGD HV	$3.19\times 10^{-2}$ ($4.43\times 10^{-4}$)+ $2.89\times 10^{-1}$ **($3.65\times 10^{-4}$)−**	$6.13\times 10^{-2}$ ($1.25\times 10^{-2}$)+ $2.72\times 10^{-1}$ ($4.06\times 10^{-3}$)+	$3.30\times 10^{-2}$ ($5.24\times 10^{-4}$)+ $2.87\times 10^{-1}$ ($3.17\times 10^{-4}$)−	$3.66\times 10^{-2}$ ($5.77\times 10^{-3}$)+ $2.84\times 10^{-1}$ ($2.45\times 10^{-3}$)+	$4.32\times 10^{-1}$ ($6.76\times 10^{-2}$)+ $1.05\times 10^{-1}$ ($2.07\times 10^{-2}$)+	NaN (0.00%)+ NaN (0.00%)+	$5.34\times 10^{-2}$ ($3.09\times 10^{-3}$)+ $2.83\times 10^{-1}$ ($5.13\times 10^{-4}$)+	$3.31\times 10^{-2}$ ($3.60\times 10^{-3}$)+ $2.88\times 10^{-1}$ ($2.40\times 10^{-3}$)−	$3.03\times 10^{-2}$ **($8.36\times 10^{-4}$)** $2.87\times 10^{-1}$ ($3.05\times 10^{-4}$)
DASCMOP8	IGD HV	$4.99\times 10^{-2}$ ($9.81\times 10^{-4}$)+ $2.05\times 10^{-1}$ ($1.29\times 10^{-3}$)+	$4.97\times 10^{-1}$ ($4.95\times 10^{-2}$)+ $1.30\times 10^{-1}$ ($2.14\times 10^{-3}$)+	$4.13\times 10^{-2}$ ($5.00\times 10^{-4}$)+ $2.06\times 10^{-1}$ ($3.46\times 10^{-4}$)+	$4.54\times 10^{-2}$ ($5.21\times 10^{-3}$)+ $2.06\times 10^{-1}$ ($9.87\times 10^{-4}$)+	$4.12\times 10^{-1}$ ($6.82\times 10^{-2}$)+ $3.23\times 10^{-2}$ ($1.45\times 10^{-2}$)+	NaN (0.00%)+ NaN (0.00%)+	$6.74\times 10^{-2}$ ($3.77\times 10^{-3}$)+ $2.03\times 10^{-1}$ ($6.94\times 10^{-4}$)+	$4.07\times 10^{-2}$ ($7.24\times 10^{-4}$)+ $2.07\times 10^{-1}$ **($4.83\times 10^{-4}$)−**	$3.87\times 10^{-2}$ **($1.07\times 10^{-3}$)** $2.07\times 10^{-1}$ ($3.21\times 10^{-4}$)
DASCMOP9	IGD HV	$5.35\times 10^{-1}$ ($3.74\times 10^{-2}$)+ $9.67\times 10^{-2}$ ($1.04\times 10^{-2}$)+	$6.04\times 10^{-1}$ ($3.70\times 10^{-2}$)+ $7.94\times 10^{-2}$ ($1.18\times 10^{-2}$)+	$4.75\times 10^{-1}$ ($3.16\times 10^{-2}$)+ $1.09\times 10^{-1}$ ($5.72\times 10^{-3}$)+	$4.13\times 10^{-1}$ ($1.87\times 10^{-1}$)+ $1.25\times 10^{-1}$ ($3.89\times 10^{-2}$)+	$4.23\times 10^{-2}$ ($1.40\times 10^{-3}$)+ $2.05\times 10^{-1}$ ($2.34\times 10^{-4}$)+	NaN (0.00%)+ NaN (0.00%)+	$3.15\times 10^{-1}$ ($2.44\times 10^{-2}$)+ $1.37\times 10^{-1}$ ($5.68\times 10^{-3}$)+	$3.99\times 10^{-1}$ ($5.74\times 10^{-2}$)+ $1.19\times 10^{-1}$ ($1.10\times 10^{-2}$)+	$3.87\times 10^{-2}$ **($8.54\times 10^{-4}$)** $2.08\times 10^{-1}$ **($2.59\times 10^{-4}$)**
+/−/=	IGD HV	9/0/0 8/1/0	9/0/0 9/0/0	8/1/0 7/2/0	9/0/0 9/0/0	9/0/0 9/0/0	9/0/0 9/0/0	9/0/0 9/0/0	9/0/0 7/2/0	– –

**Table 5:** IGD and HV results on the LIRCMOP test suite.

Problem	Metric	AGEMOEAII	ANSGAIII	DPVAPS	DRCMO	FLI	RVEAa	TriP	TSTI	MFGCMOEA
LIRCMOP1	IGD HV	$3.40\times 10^{-1}$ ($1.32\times 10^{-2}$)+ $9.98\times 10^{-2}$ ($5.16\times 10^{-3}$)+	$3.36\times 10^{-1}$ ($1.29\times 10^{-2}$)+ $9.89\times 10^{-2}$ ($4.92\times 10^{-3}$)+	$3.35\times 10^{-1}$ ($1.61\times 10^{-2}$)+ $1.02\times 10^{-1}$ ($5.71\times 10^{-3}$)+	$3.23\times 10^{-1}$ ($3.22\times 10^{-2}$)+ $1.04\times 10^{-1}$ ($1.23\times 10^{-2}$)+	$1.55\times 10^{-1}$ ($4.34\times 10^{-2}$)+ $1.61\times 10^{-1}$ ($1.59\times 10^{-2}$)+	NaN (0.00%)+ NaN (0.00%)+	$1.05\times 10^{-1}$ ($2.48\times 10^{-2}$)+ $1.78\times 10^{-1}$ ($1.01\times 10^{-2}$)+	$2.57\times 10^{-1}$ ($3.48\times 10^{-2}$)+ $1.21\times 10^{-1}$ ($1.07\times 10^{-2}$)+	$7.95\times 10^{-2}$ **($1.78\times 10^{-2}$)** $1.96\times 10^{-1}$ **($6.77\times 10^{-3}$)**
LIRCMOP2	IGD HV	$2.93\times 10^{-1}$ ($1.32\times 10^{-2}$)+ $2.12\times 10^{-1}$ ($5.57\times 10^{-3}$)+	$2.97\times 10^{-1}$ ($1.82\times 10^{-2}$)+ $2.04\times 10^{-1}$ ($1.12\times 10^{-2}$)+	$2.94\times 10^{-1}$ ($1.49\times 10^{-2}$)+ $2.11\times 10^{-1}$ ($5.91\times 10^{-3}$)+	$2.51\times 10^{-1}$ ($3.28\times 10^{-2}$)+ $2.29\times 10^{-1}$ ($1.72\times 10^{-2}$)+	$1.30\times 10^{-1}$ ($2.75\times 10^{-2}$)+ $2.92\times 10^{-1}$ ($1.44\times 10^{-2}$)+	NaN (0.00%)+ NaN (0.00%)+	$1.11\times 10^{-1}$ ($1.83\times 10^{-2}$)+ $2.98\times 10^{-1}$ ($1.15\times 10^{-2}$)+	$2.13\times 10^{-1}$ ($1.46\times 10^{-2}$)+ $2.51\times 10^{-1}$ ($7.66\times 10^{-3}$)+	$6.35\times 10^{-2}$ **($1.85\times 10^{-2}$)** $3.23\times 10^{-1}$ **($9.91\times 10^{-3}$)**
LIRCMOP3	IGD HV	$3.40\times 10^{-1}$ ($8.35\times 10^{-3}$)+ $8.90\times 10^{-2}$ ($4.69\times 10^{-3}$)+	$3.40\times 10^{-1}$ ($4.49\times 10^{-3}$)+ $8.93\times 10^{-2}$ ($3.14\times 10^{-3}$)+	$3.41\times 10^{-1}$ ($7.40\times 10^{-3}$)+ $8.93\times 10^{-2}$ ($5.01\times 10^{-3}$)+	$3.12\times 10^{-1}$ ($3.56\times 10^{-2}$)+ $9.54\times 10^{-2}$ ($1.02\times 10^{-2}$)+	$2.20\times 10^{-1}$ ($4.61\times 10^{-2}$)+ $1.25\times 10^{-1}$ ($1.55\times 10^{-2}$)+	NaN (0.00%)+ NaN (0.00%)+	$1.34\times 10^{-1}$ ($2.16\times 10^{-2}$)+ $1.51\times 10^{-1}$ ($7.40\times 10^{-3}$)+	$2.58\times 10^{-1}$ ($1.74\times 10^{-2}$)+ $1.10\times 10^{-1}$ ($5.26\times 10^{-3}$)+	$8.80\times 10^{-2}$ **($2.04\times 10^{-2}$)** $1.69\times 10^{-1}$ **($7.83\times 10^{-3}$)**
LIRCMOP4	IGD HV	$3.22\times 10^{-1}$ ($7.36\times 10^{-3}$)+ $1.74\times 10^{-1}$ ($7.61\times 10^{-3}$)+	$3.23\times 10^{-1}$ ($9.36\times 10^{-3}$)+ $1.73\times 10^{-1}$ ($8.46\times 10^{-3}$)+	$3.18\times 10^{-1}$ ($8.50\times 10^{-3}$)+ $1.77\times 10^{-1}$ ($6.89\times 10^{-3}$)+	$2.95\times 10^{-1}$ ($3.99\times 10^{-2}$)+ $1.87\times 10^{-1}$ ($2.03\times 10^{-2}$)+	$2.30\times 10^{-1}$ ($4.27\times 10^{-2}$)+ $2.18\times 10^{-1}$ ($1.49\times 10^{-2}$)+	NaN (0.00%)+ NaN (0.00%)+	$1.54\times 10^{-1}$ ($1.18\times 10^{-2}$)+ $2.48\times 10^{-1}$ ($7.04\times 10^{-3}$)+	$2.50\times 10^{-1}$ ($8.02\times 10^{-3}$)+ $2.07\times 10^{-1}$ ($7.05\times 10^{-3}$)+	$1.22\times 10^{-1}$ **($1.99\times 10^{-2}$)** $2.64\times 10^{-1}$ **($7.86\times 10^{-3}$)**
LIRCMOP5	IGD HV	$1.28\times 10^{0}$ ($2.45\times 10^{-1}$)+ $0.00\times 10^{0}$ ($0.00\times 10^{0}$)+	$1.25\times 10^{0}$ ($1.61\times 10^{-2}$)+ $0.00\times 10^{0}$ ($0.00\times 10^{0}$)+	$3.64\times 10^{-1}$ ($3.16\times 10^{-2}$)+ $1.29\times 10^{-1}$ ($1.14\times 10^{-2}$)+	$4.08\times 10^{-1}$ ($7.89\times 10^{-2}$)+ $1.12\times 10^{-1}$ ($2.79\times 10^{-2}$)+	$1.23\times 10^{0}$ ($1.22\times 10^{-3}$)+ $0.00\times 10^{0}$ ($0.00\times 10^{0}$)+	$3.99\times 10^{-1}$ ($3.28\times 10^{-2}$)+ $1.17\times 10^{-1}$ ($1.17\times 10^{-2}$)+	$8.63\times 10^{-2}$ ($6.69\times 10^{-2}$)= $2.37\times 10^{-1}$ ($2.89\times 10^{-2}$)+	$1.22\times 10^{0}$ ($2.34\times 10^{-3}$)+ $0.00\times 10^{0}$ ($0.00\times 10^{0}$)+	$6.03\times 10^{-2}$ **($1.53\times 10^{-2}$)** $2.68\times 10^{-1}$ **($6.00\times 10^{-3}$)**
LIRCMOP6	IGD HV	$1.35\times 10^{0}$ ($3.10\times 10^{-4}$)+ $0.00\times 10^{0}$ ($0.00\times 10^{0}$)+	$1.35\times 10^{0}$ ($2.70\times 10^{-4}$)+ $0.00\times 10^{0}$ ($0.00\times 10^{0}$)+	$4.02\times 10^{-1}$ ($4.48\times 10^{-2}$)+ $1.01\times 10^{-1}$ ($2.26\times 10^{-3}$)+	$4.72\times 10^{-1}$ ($1.20\times 10^{-1}$)+ $8.67\times 10^{-2}$ ($1.10\times 10^{-2}$)+	$1.35\times 10^{0}$ ($1.25\times 10^{-4}$)+ $0.00\times 10^{0}$ ($0.00\times 10^{0}$)+	$5.30\times 10^{-1}$ ($6.80\times 10^{-2}$)+ $8.72\times 10^{-2}$ ($5.76\times 10^{-3}$)+	$2.22\times 10^{-1}$ ($4.40\times 10^{-2}$)+ $1.46\times 10^{-1}$ ($1.42\times 10^{-2}$)+	$1.35\times 10^{0}$ ($1.35\times 10^{-4}$)+ $0.00\times 10^{0}$ ($0.00\times 10^{0}$)+	$2.61\times 10^{-2}$ **($5.36\times 10^{-3}$)** $1.86\times 10^{-1}$ **($2.75\times 10^{-3}$)**
LIRCMOP7	IGD HV	$1.68\times 10^{0}$ ($1.38\times 10^{-4}$)+ $0.00\times 10^{0}$ ($0.00\times 10^{0}$)+	$1.68\times 10^{0}$ ($4.88\times 10^{-4}$)+ $0.00\times 10^{0}$ ($0.00\times 10^{0}$)+	$1.70\times 10^{-1}$ ($1.93\times 10^{-2}$)+ $2.34\times 10^{-1}$ ($5.11\times 10^{-3}$)+	$1.89\times 10^{-1}$ ($3.48\times 10^{-2}$)+ $2.29\times 10^{-1}$ ($9.84\times 10^{-3}$)+	$1.75\times 10^{-1}$ ($2.38\times 10^{-2}$)+ $2.32\times 10^{-1}$ ($6.05\times 10^{-3}$)+	NaN (0.08%)+ NaN (0.08%)+	$1.43\times 10^{-1}$ ($1.17\times 10^{-2}$)+ $2.41\times 10^{-1}$ ($3.27\times 10^{-3}$)+	$2.49\times 10^{-1}$ ($2.72\times 10^{-1}$)+ $2.20\times 10^{-1}$ ($4.19\times 10^{-2}$)+	$7.36\times 10^{-3}$ **($2.17\times 10^{-4}$)** $2.94\times 10^{-1}$ **($1.84\times 10^{-4}$)**
LIRCMOP8	IGD HV	$1.68\times 10^{0}$ ($3.53\times 10^{-4}$)+ $0.00\times 10^{0}$ ($0.00\times 10^{0}$)+	$1.68\times 10^{0}$ ($3.61\times 10^{-4}$)+ $0.00\times 10^{0}$ ($0.00\times 10^{0}$)+	$2.56\times 10^{-1}$ ($2.51\times 10^{-2}$)+ $2.21\times 10^{-1}$ ($1.52\times 10^{-3}$)+	$3.13\times 10^{-1}$ ($7.37\times 10^{-2}$)+ $2.15\times 10^{-1}$ ($7.15\times 10^{-3}$)+	$2.17\times 10^{-1}$ ($2.77\times 10^{-2}$)+ $2.25\times 10^{-1}$ ($3.54\times 10^{-3}$)+	NaN (0.03%)+ NaN (0.03%)+	$2.00\times 10^{-1}$ ($1.69\times 10^{-2}$)+ $2.30\times 10^{-1}$ ($2.41\times 10^{-3}$)+	$6.10\times 10^{-1}$ ($5.45\times 10^{-1}$)+ $1.70\times 10^{-1}$ ($8.68\times 10^{-2}$)+	$7.24\times 10^{-3}$ **($1.16\times 10^{-4}$)** $2.95\times 10^{-1}$ **($1.20\times 10^{-4}$)**
LIRCMOP9	IGD HV	$1.07\times 10^{0}$ ($1.98\times 10^{-2}$)+ $8.39\times 10^{-2}$ ($3.57\times 10^{-3}$)+	$1.07\times 10^{0}$ ($2.00\times 10^{-2}$)+ $8.40\times 10^{-2}$ ($4.16\times 10^{-3}$)+	$6.46\times 10^{-1}$ ($5.31\times 10^{-2}$)+ $2.95\times 10^{-1}$ ($2.59\times 10^{-2}$)+	$5.87\times 10^{-1}$ ($9.29\times 10^{-2}$)+ $3.04\times 10^{-1}$ ($5.39\times 10^{-2}$)+	$4.59\times 10^{-1}$ ($8.68\times 10^{-2}$)+ $3.71\times 10^{-1}$ ($4.63\times 10^{-2}$)+	$6.22\times 10^{-1}$ ($2.40\times 10^{-1}$)+ $3.07\times 10^{-1}$ ($4.88\times 10^{-2}$)+	$5.35\times 10^{-1}$ ($8.65\times 10^{-2}$)+ $3.37\times 10^{-1}$ ($4.67\times 10^{-2}$)+	$6.73\times 10^{-1}$ ($3.94\times 10^{-2}$)+ $2.36\times 10^{-1}$ ($1.87\times 10^{-2}$)+	$3.11\times 10^{-1}$ **($7.80\times 10^{-2}$)** $4.59\times 10^{-1}$ **($2.46\times 10^{-2}$)**
LIRCMOP10	IGD HV	$1.03\times 10^{0}$ ($7.48\times 10^{-2}$)+ $5.26\times 10^{-2}$ ($9.89\times 10^{-4}$)+	$1.04\times 10^{0}$ ($2.90\times 10^{-2}$)+ $5.17\times 10^{-2}$ ($3.38\times 10^{-4}$)+	$2.48\times 10^{-1}$ ($4.78\times 10^{-2}$)+ $5.70\times 10^{-1}$ ($2.69\times 10^{-2}$)+	$5.67\times 10^{-1}$ ($1.96\times 10^{-1}$)+ $3.00\times 10^{-1}$ ($1.64\times 10^{-1}$)+	$3.39\times 10^{-1}$ ($1.74\times 10^{-1}$)+ $5.05\times 10^{-1}$ ($1.45\times 10^{-1}$)+	$2.17\times 10^{-1}$ ($9.25\times 10^{-2}$)+ $5.81\times 10^{-1}$ ($6.43\times 10^{-2}$)+	$4.12\times 10^{-1}$ ($4.41\times 10^{-4}$)+ $4.89\times 10^{-1}$ ($1.26\times 10^{-3}$)+	$8.96\times 10^{-1}$ ($9.21\times 10^{-2}$)+ $9.50\times 10^{-2}$ ($4.41\times 10^{-2}$)+	$1.61\times 10^{-1}$ **($3.86\times 10^{-2}$)** $6.34\times 10^{-1}$ **($2.03\times 10^{-2}$)**
LIRCMOP11	IGD HV	$8.70\times 10^{-1}$ ($7.00\times 10^{-2}$)+ $1.74\times 10^{-1}$ ($1.70\times 10^{-2}$)+	$9.78\times 10^{-1}$ ($2.99\times 10^{-2}$)+ $1.59\times 10^{-1}$ ($1.38\times 10^{-2}$)+	$1.15\times 10^{-1}$ ($4.39\times 10^{-2}$)+ $6.28\times 10^{-1}$ ($3.07\times 10^{-2}$)+	$5.28\times 10^{-1}$ ($1.41\times 10^{-1}$)+ $3.27\times 10^{-1}$ ($1.17\times 10^{-1}$)+	$3.63\times 10^{-1}$ ($1.28\times 10^{-1}$)+ $4.48\times 10^{-1}$ ($9.75\times 10^{-2}$)+	$5.03\times 10^{-1}$ ($2.23\times 10^{-1}$)+ $5.05\times 10^{-1}$ ($6.71\times 10^{-2}$)+	$1.48\times 10^{-1}$ ($6.80\times 10^{-3}$)+ $6.02\times 10^{-1}$ ($1.06\times 10^{-2}$)+	$7.56\times 10^{-1}$ ($7.35\times 10^{-3}$)+ $2.26\times 10^{-1}$ ($2.67\times 10^{-3}$)+	$4.69\times 10^{-2}$ **($3.28\times 10^{-2}$)** $6.73\times 10^{-1}$ **($1.49\times 10^{-2}$)**
LIRCMOP12	IGD HV	$9.63\times 10^{-1}$ ($2.91\times 10^{-2}$)+ $1.85\times 10^{-1}$ ($1.23\times 10^{-2}$)+	$9.92\times 10^{-1}$ ($4.73\times 10^{-2}$)+ $1.71\times 10^{-1}$ ($2.08\times 10^{-2}$)+	$3.20\times 10^{-1}$ ($5.09\times 10^{-2}$)+ $4.55\times 10^{-1}$ ($1.98\times 10^{-2}$)+	$3.60\times 10^{-1}$ ($7.01\times 10^{-2}$)+ $4.21\times 10^{-1}$ ($5.03\times 10^{-2}$)+	$2.24\times 10^{-1}$ ($6.60\times 10^{-2}$)+ $5.10\times 10^{-1}$ ($2.87\times 10^{-2}$)+	$1.31\times 10^{0}$ ($9.13\times 10^{-1}$)+ $2.86\times 10^{-1}$ ($1.46\times 10^{-1}$)+	$2.26\times 10^{-1}$ ($4.97\times 10^{-2}$)+ $5.09\times 10^{-1}$ ($2.47\times 10^{-2}$)+	$4.43\times 10^{-1}$ ($1.31\times 10^{-1}$)+ $3.96\times 10^{-1}$ ($5.75\times 10^{-2}$)+	$1.23\times 10^{-1}$ **($2.20\times 10^{-2}$)** $5.62\times 10^{-1}$ **($1.24\times 10^{-2}$)**
LIRCMOP13	IGD HV	$1.31\times 10^{0}$ ($1.65\times 10^{-3}$)+ $4.16\times 10^{-4}$ ($4.70\times 10^{-5}$)+	$1.32\times 10^{0}$ ($2.60\times 10^{-3}$)+ $2.36\times 10^{-6}$ ($8.99\times 10^{-6}$)+	$9.10\times 10^{-2}$ **($5.72\times 10^{-4}$)−** $5.60\times 10^{-1}$ **($6.22\times 10^{-4}$)−**	$1.03\times 10^{-1}$ ($1.65\times 10^{-3}$)− $5.34\times 10^{-1}$ ($2.14\times 10^{-3}$)−	$9.98\times 10^{-2}$ ($1.03\times 10^{-3}$)− $5.52\times 10^{-1}$ ($6.21\times 10^{-4}$)−	$9.46\times 10^{-2}$ ($3.24\times 10^{-4}$)− $5.57\times 10^{-1}$ ($4.98\times 10^{-4}$)−	$1.05\times 10^{-1}$ ($9.13\times 10^{-4}$)= $5.41\times 10^{-1}$ ($1.58\times 10^{-3}$)−	$1.32\times 10^{0}$ ($8.98\times 10^{-4}$)+ $2.49\times 10^{-6}$ ($1.36\times 10^{-5}$)+	$1.06\times 10^{-1}$ ($1.26\times 10^{-3}$) $5.30\times 10^{-1}$ ($1.65\times 10^{-3}$)
LIRCMOP14	IGD HV	$1.26\times 10^{0}$ ($1.46\times 10^{-3}$)+ $9.41\times 10^{-4}$ ($1.78\times 10^{-4}$)+	$1.28\times 10^{0}$ ($2.91\times 10^{-3}$)+ $9.70\times 10^{-5}$ ($1.61\times 10^{-4}$)+	$9.35\times 10^{-2}$ **($5.04\times 10^{-4}$)−** $5.59\times 10^{-1}$ **($7.30\times 10^{-4}$)−**	$1.29\times 10^{-1}$ ($9.75\times 10^{-2}$)+ $5.29\times 10^{-1}$ ($6.39\times 10^{-2}$)=	$1.02\times 10^{-1}$ ($1.29\times 10^{-3}$)+ $5.55\times 10^{-1}$ ($7.07\times 10^{-4}$)−	$1.74\times 10^{-1}$ ($1.02\times 10^{-2}$)+ $5.03\times 10^{-1}$ ($5.65\times 10^{-3}$)+	$1.05\times 10^{-1}$ ($1.15\times 10^{-3}$)+ $5.46\times 10^{-1}$ ($1.04\times 10^{-3}$)+	$1.27\times 10^{0}$ ($8.41\times 10^{-4}$)+ $1.92\times 10^{-4}$ ($1.63\times 10^{-4}$)+	$9.73\times 10^{-2}$ ($8.19\times 10^{-4}$) $5.52\times 10^{-1}$ ($1.17\times 10^{-3}$)
+/−/=	IGD HV	14/0/0 14/0/0	14/0/0 14/0/0	12/2/0 12/2/0	13/1/0 12/1/1	13/1/0 12/2/0	13/1/0 13/1/0	12/0/2 13/1/0	14/0/0 14/0/0	– –

**Table 6:** IGD and HV results on the MW test suite.

Problem	Metric	AGEMOEAII	ANSGAIII	DPVAPS	DRCMO	FLI	RVEAa	TriP	TSTI	MFGCMOEA
MW1	IGD HV	NaN (13.33%)+ NaN (13.33%)+	NaN (0.00%)+ NaN (0.00%)+	NaN (46.67%)+ NaN (46.67%)+	$3.97\times 10^{-1}$ ($2.61\times 10^{-1}$)+ $1.78\times 10^{-1}$ ($1.70\times 10^{-1}$)+	$1.26\times 10^{-1}$ ($1.19\times 10^{-1}$)+ $3.38\times 10^{-1}$ ($9.46\times 10^{-2}$)+	NaN (0.00%)+ NaN (0.00%)+	NaN (30.00%)+ NaN (30.00%)+	NaN (36.70%)+ NaN (36.70%)+	$3.70\times 10^{-3}$ **($1.82\times 10^{-3}$)** $4.85\times 10^{-1}$ **($3.68\times 10^{-3}$)**
MW2	IGD HV	$1.40\times 10^{-1}$ ($5.20\times 10^{-2}$)+ $3.98\times 10^{-1}$ ($5.82\times 10^{-2}$)+	$1.01\times 10^{-1}$ ($3.06\times 10^{-2}$)+ $4.43\times 10^{-1}$ ($3.66\times 10^{-2}$)+	$1.01\times 10^{-1}$ ($4.12\times 10^{-2}$)+ $4.44\times 10^{-1}$ ($4.83\times 10^{-2}$)+	$3.61\times 10^{-1}$ ($3.48\times 10^{-2}$)+ $1.86\times 10^{-1}$ ($2.06\times 10^{-2}$)+	$3.38\times 10^{-2}$ **($2.20\times 10^{-3}$)−** $5.33\times 10^{-1}$ **($3.20\times 10^{-3}$)−**	$5.83\times 10^{-2}$ ($1.49\times 10^{-2}$)= $4.97\times 10^{-1}$ ($2.06\times 10^{-2}$)=	$9.98\times 10^{-2}$ ($4.52\times 10^{-2}$)+ $4.47\times 10^{-1}$ ($5.35\times 10^{-2}$)+	$1.17\times 10^{-1}$ ($4.52\times 10^{-2}$)+ $4.26\times 10^{-1}$ ($5.31\times 10^{-2}$)+	$6.28\times 10^{-2}$ ($1.17\times 10^{-2}$) $4.93\times 10^{-1}$ ($1.59\times 10^{-2}$)
MW3	IGD HV	$9.19\times 10^{-1}$ ($4.79\times 10^{-8}$)+ $0.00\times 10^{0}$ ($0.00\times 10^{0}$)+	$9.19\times 10^{-1}$ ($5.96\times 10^{-8}$)+ $0.00\times 10^{0}$ ($0.00\times 10^{0}$)+	$8.92\times 10^{-3}$ ($1.00\times 10^{-3}$)+ $5.37\times 10^{-1}$ ($1.53\times 10^{-3}$)+	$1.65\times 10^{-2}$ ($3.33\times 10^{-3}$)+ $5.25\times 10^{-1}$ ($6.20\times 10^{-3}$)+	$8.65\times 10^{-3}$ ($4.44\times 10^{-4}$)+ $5.37\times 10^{-1}$ ($7.35\times 10^{-4}$)+	$4.99\times 10^{-2}$ ($1.09\times 10^{-3}$)+ $5.30\times 10^{-1}$ ($2.19\times 10^{-3}$)+	$9.23\times 10^{-3}$ ($7.89\times 10^{-4}$)+ $5.37\times 10^{-1}$ ($1.20\times 10^{-3}$)+	$1.54\times 10^{-1}$ ($1.63\times 10^{-1}$)+ $4.46\times 10^{-1}$ ($9.72\times 10^{-2}$)+	$6.44\times 10^{-3}$ **($2.54\times 10^{-4}$)** $5.41\times 10^{-1}$ **($3.70\times 10^{-4}$)**
MW4	IGD HV	$7.32\times 10^{-2}$ ($9.62\times 10^{-2}$)− $7.94\times 10^{-1}$ ($1.16\times 10^{-1}$)−	$9.45\times 10^{-2}$ ($9.80\times 10^{-2}$)= $7.61\times 10^{-1}$ ($1.23\times 10^{-1}$)=	$7.59\times 10^{-2}$ ($1.00\times 10^{-1}$)− $7.98\times 10^{-1}$ ($1.22\times 10^{-1}$)−	$1.85\times 10^{-1}$ ($2.12\times 10^{-1}$)+ $6.66\times 10^{-1}$ ($2.17\times 10^{-1}$)+	$1.78\times 10^{-1}$ ($8.19\times 10^{-2}$)+ $6.34\times 10^{-1}$ ($1.06\times 10^{-1}$)+	$4.40\times 10^{-2}$ **($8.48\times 10^{-4}$)−** $8.36\times 10^{-1}$ **($1.18\times 10^{-3}$)−**	$5.96\times 10^{-2}$ ($4.89\times 10^{-2}$)= $8.13\times 10^{-1}$ ($7.34\times 10^{-2}$)=	$8.82\times 10^{-2}$ ($1.27\times 10^{-1}$)− $7.84\times 10^{-1}$ ($1.37\times 10^{-1}$)−	$4.89\times 10^{-2}$ ($9.48\times 10^{-4}$) $8.27\times 10^{-1}$ ($1.42\times 10^{-3}$)
MW5	IGD HV	$7.40\times 10^{-1}$ ($8.04\times 10^{-3}$)+ $9.06\times 10^{-2}$ ($8.95\times 10^{-4}$)+	$7.42\times 10^{-1}$ ($8.98\times 10^{-3}$)+ $8.96\times 10^{-2}$ ($6.79\times 10^{-3}$)+	$1.09\times 10^{-1}$ ($1.61\times 10^{-1}$)+ $2.75\times 10^{-1}$ ($5.78\times 10^{-2}$)+	$7.73\times 10^{-1}$ ($6.08\times 10^{-2}$)+ $7.38\times 10^{-2}$ ($3.12\times 10^{-2}$)+	$1.58\times 10^{-1}$ ($2.44\times 10^{-1}$)+ $2.18\times 10^{-1}$ ($6.63\times 10^{-2}$)+	NaN (0.25%)+ NaN (0.25%)+	$4.94\times 10^{-2}$ ($1.39\times 10^{-1}$)+ $2.96\times 10^{-1}$ ($6.09\times 10^{-2}$)+	$7.39\times 10^{-1}$ ($1.27\times 10^{-2}$)+ $9.03\times 10^{-2}$ ($2.78\times 10^{-3}$)+	$2.91\times 10^{-3}$ **($5.24\times 10^{-4}$)** $3.22\times 10^{-1}$ **($3.75\times 10^{-4}$)**
MW6	IGD HV	$5.79\times 10^{-1}$ ($9.28\times 10^{-2}$)+ $7.23\times 10^{-2}$ ($3.25\times 10^{-2}$)+	$5.46\times 10^{-1}$ ($6.99\times 10^{-2}$)+ $8.84\times 10^{-2}$ ($3.25\times 10^{-2}$)+	$5.19\times 10^{-1}$ ($6.32\times 10^{-2}$)+ $1.08\times 10^{-1}$ ($3.30\times 10^{-2}$)+	$1.18\times 10^{0}$ ($1.07\times 10^{-1}$)+ $0.00\times 10^{0}$ ($0.00\times 10^{0}$)+	$5.77\times 10^{-2}$ **($1.37\times 10^{-2}$)−** $2.55\times 10^{-1}$ **($8.51\times 10^{-3}$)−**	$1.11\times 10^{-1}$ ($8.58\times 10^{-2}$)+ $2.23\times 10^{-1}$ ($3.36\times 10^{-2}$)+	$1.37\times 10^{-1}$ ($1.76\times 10^{-1}$)= $2.29\times 10^{-1}$ ($5.97\times 10^{-2}$)=	$5.75\times 10^{-1}$ ($1.23\times 10^{-1}$)+ $8.39\times 10^{-2}$ ($4.28\times 10^{-2}$)+	$8.30\times 10^{-2}$ ($3.05\times 10^{-2}$) $2.40\times 10^{-1}$ ($2.24\times 10^{-2}$)
MW7	IGD HV	$4.51\times 10^{-1}$ ($4.04\times 10^{-4}$)+ $2.44\times 10^{-1}$ ($8.23\times 10^{-4}$)+	$4.50\times 10^{-1}$ ($1.73\times 10^{-3}$)+ $2.38\times 10^{-1}$ ($4.49\times 10^{-3}$)+	$6.74\times 10^{-3}$ **($4.38\times 10^{-4}$)=** $4.08\times 10^{-1}$ ($8.82\times 10^{-4}$)+	$1.11\times 10^{-2}$ ($6.71\times 10^{-4}$)+ $4.02\times 10^{-1}$ ($9.47\times 10^{-4}$)+	$1.02\times 10^{-2}$ ($6.56\times 10^{-4}$)+ $4.02\times 10^{-1}$ ($1.23\times 10^{-3}$)+	$2.01\times 10^{-1}$ ($1.25\times 10^{-3}$)+ $3.39\times 10^{-1}$ ($2.16\times 10^{-3}$)+	$6.85\times 10^{-3}$ ($3.73\times 10^{-4}$)= $4.07\times 10^{-1}$ ($7.46\times 10^{-4}$)+	$5.16\times 10^{-2}$ ($2.68\times 10^{-4}$)+ $4.00\times 10^{-1}$ ($1.68\times 10^{-3}$)+	$6.78\times 10^{-3}$ ($3.81\times 10^{-4}$) $4.09\times 10^{-1}$ **($8.27\times 10^{-4}$)**
MW8	IGD HV	$2.06\times 10^{-1}$ ($1.28\times 10^{-1}$)+ $3.19\times 10^{-1}$ ($8.55\times 10^{-2}$)+	$4.91\times 10^{-1}$ ($8.42\times 10^{-2}$)+ $2.17\times 10^{-1}$ ($4.53\times 10^{-2}$)+	$1.17\times 10^{-1}$ ($3.83\times 10^{-2}$)+ $3.86\times 10^{-1}$ ($6.40\times 10^{-2}$)+	$4.93\times 10^{-1}$ ($3.81\times 10^{-1}$)+ $1.40\times 10^{-1}$ ($9.61\times 10^{-2}$)+	$8.50\times 10^{-2}$ ($3.86\times 10^{-3}$)+ $4.44\times 10^{-1}$ ($7.30\times 10^{-3}$)+	$7.15\times 10^{-2}$ ($1.25\times 10^{-2}$)− $4.74\times 10^{-1}$ ($3.00\times 10^{-2}$)−	$6.88\times 10^{-2}$ **($1.01\times 10^{-2}$)−** $4.77\times 10^{-1}$ **($2.07\times 10^{-2}$)−**	$1.48\times 10^{-1}$ ($5.28\times 10^{-2}$)+ $3.40\times 10^{-1}$ ($7.42\times 10^{-2}$)+	$7.65\times 10^{-2}$ ($8.13\times 10^{-3}$) $4.60\times 10^{-1}$ ($1.67\times 10^{-2}$)
MW9	IGD HV	$7.09\times 10^{-1}$ ($6.99\times 10^{-3}$)+ $0.00\times 10^{0}$ ($0.00\times 10^{0}$)+	$7.11\times 10^{-1}$ ($9.68\times 10^{-3}$)+ $0.00\times 10^{0}$ ($0.00\times 10^{0}$)+	$7.14\times 10^{-1}$ ($9.81\times 10^{-4}$)+ $0.00\times 10^{0}$ ($0.00\times 10^{0}$)+	$7.29\times 10^{-1}$ ($2.96\times 10^{-2}$)+ $0.00\times 10^{0}$ ($0.00\times 10^{0}$)+	$7.88\times 10^{-2}$ ($1.61\times 10^{-1}$)+ $3.31\times 10^{-1}$ ($6.36\times 10^{-2}$)+	$6.92\times 10^{-1}$ ($8.10\times 10^{-2}$)+ $3.89\times 10^{-3}$ ($1.71\times 10^{-2}$)+	$7.30\times 10^{-1}$ ($5.66\times 10^{-3}$)+ $0.00\times 10^{0}$ ($0.00\times 10^{0}$)+	$7.16\times 10^{-1}$ ($1.05\times 10^{-2}$)+ $0.00\times 10^{0}$ ($0.00\times 10^{0}$)+	$5.83\times 10^{-3}$ **($8.49\times 10^{-4}$)** $3.94\times 10^{-1}$ **($2.74\times 10^{-3}$)**
MW10	IGD HV	$6.34\times 10^{-1}$ ($4.68\times 10^{-2}$)+ $1.19\times 10^{-1}$ ($2.34\times 10^{-2}$)+	$6.11\times 10^{-1}$ ($4.06\times 10^{-2}$)+ $1.32\times 10^{-1}$ ($2.08\times 10^{-2}$)+	$3.77\times 10^{-1}$ ($1.43\times 10^{-1}$)+ $2.33\times 10^{-1}$ ($6.25\times 10^{-2}$)+	$6.28\times 10^{-1}$ ($3.61\times 10^{-2}$)+ $1.21\times 10^{-1}$ ($1.77\times 10^{-2}$)+	$1.89\times 10^{-1}$ ($7.43\times 10^{-2}$)= $3.25\times 10^{-1}$ ($3.09\times 10^{-2}$)=	$3.11\times 10^{-1}$ ($1.78\times 10^{-1}$)+ $2.64\times 10^{-1}$ ($8.36\times 10^{-2}$)+	$2.39\times 10^{-1}$ ($1.65\times 10^{-1}$)= $3.02\times 10^{-1}$ ($7.61\times 10^{-2}$)=	$5.79\times 10^{-1}$ ($9.61\times 10^{-2}$)+ $1.44\times 10^{-1}$ ($4.19\times 10^{-2}$)+	$1.67\times 10^{-1}$ **($4.10\times 10^{-2}$)** $3.36\times 10^{-1}$ **($2.32\times 10^{-2}$)**
MW11	IGD HV	$6.97\times 10^{-1}$ ($1.04\times 10^{-3}$)+ $2.73\times 10^{-1}$ ($2.86\times 10^{-4}$)+	$8.13\times 10^{-1}$ ($7.65\times 10^{-2}$)+ $2.56\times 10^{-1}$ ($9.76\times 10^{-3}$)+	$6.58\times 10^{-3}$ ($3.49\times 10^{-4}$)+ $4.47\times 10^{-1}$ ($3.61\times 10^{-4}$)+	$7.89\times 10^{-3}$ ($4.82\times 10^{-4}$)+ $4.46\times 10^{-1}$ ($3.44\times 10^{-4}$)+	$6.91\times 10^{-3}$ ($2.31\times 10^{-4}$)+ $4.47\times 10^{-1}$ ($2.02\times 10^{-4}$)+	NaN (0.00%)+ NaN (0.00%)+	$6.25\times 10^{-3}$ ($9.25\times 10^{-5}$)+ $4.47\times 10^{-1}$ ($1.62\times 10^{-4}$)+	$3.68\times 10^{-1}$ ($3.45\times 10^{-1}$)+ $3.55\times 10^{-1}$ ($8.74\times 10^{-2}$)+	$6.18\times 10^{-3}$ **($1.74\times 10^{-4}$)** $4.48\times 10^{-1}$ **($1.94\times 10^{-4}$)**
MW12	IGD HV	$7.92\times 10^{-1}$ ($5.63\times 10^{-2}$)+ $0.00\times 10^{0}$ ($0.00\times 10^{0}$)+	$8.13\times 10^{-1}$ ($7.84\times 10^{-2}$)+ $0.00\times 10^{0}$ ($0.00\times 10^{0}$)+	$7.79\times 10^{-1}$ ($2.34\times 10^{-2}$)+ $0.00\times 10^{0}$ ($0.00\times 10^{0}$)+	$8.08\times 10^{-1}$ ($7.31\times 10^{-2}$)+ $0.00\times 10^{0}$ ($0.00\times 10^{0}$)+	$1.10\times 10^{-1}$ ($1.36\times 10^{-1}$)+ $4.88\times 10^{-1}$ ($1.00\times 10^{-1}$)+	NaN (26.57%)+ NaN (26.57%)+	$8.11\times 10^{-1}$ ($1.00\times 10^{-1}$)+ $0.00\times 10^{0}$ ($0.00\times 10^{0}$)+	$7.76\times 10^{-1}$ ($9.89\times 10^{-3}$)+ $0.00\times 10^{0}$ ($0.00\times 10^{0}$)+	$4.74\times 10^{-3}$ **($8.63\times 10^{-5}$)** $6.05\times 10^{-1}$ **($2.46\times 10^{-4}$)**
MW13	IGD HV	$1.13\times 10^{0}$ ($3.15\times 10^{-1}$)+ $1.93\times 10^{-1}$ ($4.13\times 10^{-2}$)+	$1.21\times 10^{0}$ ($2.64\times 10^{-1}$)+ $1.89\times 10^{-1}$ ($2.65\times 10^{-2}$)+	$2.75\times 10^{-1}$ ($1.04\times 10^{-1}$)+ $3.19\times 10^{-1}$ ($5.68\times 10^{-2}$)+	$1.74\times 10^{0}$ ($1.38\times 10^{-1}$)+ $0.00\times 10^{0}$ ($0.00\times 10^{0}$)+	$4.53\times 10^{-1}$ ($3.42\times 10^{-1}$)+ $2.80\times 10^{-1}$ ($1.06\times 10^{-1}$)+	$3.71\times 10^{-1}$ ($4.10\times 10^{-2}$)+ $2.68\times 10^{-1}$ ($1.85\times 10^{-2}$)+	$1.34\times 10^{-1}$ **($2.41\times 10^{-2}$)−** $4.06\times 10^{-1}$ **($1.74\times 10^{-2}$)−**	$4.58\times 10^{-1}$ ($1.59\times 10^{-1}$)+ $2.76\times 10^{-1}$ ($5.76\times 10^{-2}$)+	$1.45\times 10^{-1}$ ($1.57\times 10^{-2}$) $3.94\times 10^{-1}$ ($1.35\times 10^{-2}$)
MW14	IGD HV	$2.36\times 10^{-1}$ ($6.11\times 10^{-2}$)− $4.33\times 10^{-1}$ ($2.20\times 10^{-2}$)−	$6.33\times 10^{-1}$ ($6.22\times 10^{-2}$)+ $2.09\times 10^{-1}$ ($2.91\times 10^{-2}$)+	$3.45\times 10^{-1}$ ($8.19\times 10^{-2}$)= $3.86\times 10^{-1}$ ($3.91\times 10^{-2}$)=	$4.46\times 10^{-1}$ ($2.62\times 10^{-2}$)+ $3.27\times 10^{-1}$ ($1.47\times 10^{-2}$)+	$4.58\times 10^{-1}$ ($4.16\times 10^{-2}$)+ $3.15\times 10^{-1}$ ($2.10\times 10^{-2}$)+	$1.15\times 10^{-1}$ **($3.81\times 10^{-2}$)−** $4.64\times 10^{-1}$ **($6.94\times 10^{-3}$)−**	$6.31\times 10^{-1}$ ($5.22\times 10^{-2}$)+ $2.22\times 10^{-1}$ ($1.41\times 10^{-2}$)+	$2.29\times 10^{-1}$ ($5.49\times 10^{-2}$)− $4.32\times 10^{-1}$ ($1.89\times 10^{-2}$)−	$3.08\times 10^{-1}$ ($4.76\times 10^{-2}$) $3.93\times 10^{-1}$ ($1.66\times 10^{-2}$)
+/−/=	IGD HV	12/2/0 12/2/0	13/0/1 13/0/1	11/1/2 12/1/1	14/0/0 14/0/0	11/2/1 11/2/1	10/3/1 10/3/1	8/2/4 9/2/3	12/2/0 12/2/0	– –

**Table 7:** Runtime comparison of the nine algorithms on the four benchmark suites. Values are reported as mean (standard deviation).

Algorithm	CF	DASCMOP	LIRCMOP	MW
AGEMOEAII	58.55 (4.53)	71.86 (6.78)	264.81 (21.25)	164.77 (4.88)
ANSGAIII	69.43 (7.95)	41.49 (15.40)	238.29 (100.58)	94.72 (16.16)
DPVAPS	1475.23 (93.31)	581.82 (31.99)	1702.11 (43.94)	1707.57 (108.25)
DRCMO	544.13 (26.60)	507.02 (22.89)	1120.32 (18.75)	743.92 (37.01)
FLI	166.50 (21.84)	88.46 (9.21)	229.79 (32.48)	136.83 (4.15)
RVEAa	96.37 (3.45)	92.82 (2.01)	118.85 (2.58)	151.33 (3.82)
TriP	698.82 (22.10)	622.99 (23.29)	856.97 (12.68)	871.38 (18.23)
TSTI	143.04 (7.59)	143.26 (4.64)	206.19 (4.06)	275.76 (10.02)
MFGCMOEA	57.62 (7.23)	124.53 (7.70)	165.83 (2.40)	168.86 (4.02)

**Table 8:** Wilcoxon signed-rank test results for IGD between MFGCMOEA and the eight competitors.

MFGCMOEA VS	$R^{+}$	$R^{-}$	*p*	Level = 0.05
AGEMOEAII	1023	12	$5.98287\times 10^{-9}$	YES
ANSGAIII	1033	2	$3.06652\times 10^{-9}$	YES
DPVAPS	1074	54	$3.49146\times 10^{-8}$	YES
DRCMO	1034	1	$2.86631\times 10^{-9}$	YES
FLI	1083	45	$2.04583\times 10^{-8}$	YES
RVEAa	770	358	$1.48288\times 10^{-2}$	YES
TriP	1056	72	$9.90397\times 10^{-8}$	YES
TSTI	1019	16	$7.78944\times 10^{-9}$	YES

**Table 9:** Wilcoxon signed-rank test results for HV between MFGCMOEA and the eight competitors.

MFGCMOEA VS	$R^{+}$	$R^{-}$	*p*	Level = 0.05
AGEMOEAII	1026	9	$4.90226\times 10^{-9}$	YES
ANSGAIII	1035	0	$2.67883\times 10^{-9}$	YES
DPVAPS	1026	102	$5.20656\times 10^{-7}$	YES
DRCMO	1031	4	$3.50857\times 10^{-9}$	YES
FLI	987	94	$5.50370\times 10^{-7}$	YES
RVEAa	1045	83	$1.84077\times 10^{-7}$	YES
TriP	986	142	$4.09072\times 10^{-6}$	YES
TSTI	1023	12	$5.98287\times 10^{-9}$	YES

**Table 10:** Across-problem Holm-corrected comparisons and effect sizes between MFGCMOEA and the eight competitors.

	IGD		HV	
Algorithm	$p_{\mathrm{Holm}}$	$r_{\mathrm{rb}}$	$p_{\mathrm{Holm}}$	$r_{\mathrm{rb}}$
AGEMOEAII	$7.08\times 10^{-5}$	0.983	$6.64\times 10^{-5}$	0.989
ANSGAIII	$6.64\times 10^{-5}$	1.000	$6.64\times 10^{-5}$	1.000
DPVAPS	$9.40\times 10^{-5}$	0.949	$1.18\times 10^{-4}$	0.937
DRCMO	$6.64\times 10^{-5}$	0.994	$6.64\times 10^{-5}$	0.994
FLI	$2.64\times 10^{-4}$	0.858	$3.24\times 10^{-4}$	0.846
RVEA_*a*_	$2.64\times 10^{-4}$	0.840	$3.24\times 10^{-4}$	0.835
TriP	$1.90\times 10^{-4}$	0.897	$2.90\times 10^{-4}$	0.875
TSTI	$8.35\times 10^{-5}$	0.966	$6.64\times 10^{-5}$	0.989

**Table 11:** Ablation and mechanism-analysis variants of MFGCMOEA.

Variant	Modified Mechanism	Modification and Purpose
MFGCMOEA	Complete algorithm	Retains all proposed mechanisms and the original coupled variation operators as the common reference.
A	No morphogen propagation	Uses intrinsic objective-channel activations without neighborhood propagation to assess inter-individual morphogen signaling.
B	No developmental buffering	Sets the developmental buffer to zero so that constraint disturbance directly affects the developmental phenotype.
C	Scalar developmental phenotype	Replaces the vector-valued developmental phenotype by the arithmetic mean of its channels to assess objective-specific information preservation.
D	No contact inhibition	Truncates the critical front according to the mean developmental deficit without propagation-similarity-based contact inhibition.
E	Fixed resource allocation	Assigns *N* offspring to each search process throughout evolution while maintaining the total offspring budget at 2N.
F	No homeostatic basal growth	Removes the shared basal-growth term while retaining evidence-driven dynamic allocation.
G	No propagation and no buffering	Simultaneously removes morphogen propagation and developmental buffering to examine their joint contribution.
H	Sparse propagation kernel	Retains only propagation links satisfying $d_{ij}\leq h$ using the original adaptive scale *h* to examine propagation sparsity.
I	Arithmetic-mean developmental buffer	Replaces the proposed geometric developmental buffer by the arithmetic mean of the propagated objective-channel activations.
J	Common variation operator	Uses DE/rand/1 with common settings in both offspring streams to examine the contribution of operator heterogeneity.

**Table 12:** Overall ablation statistics of MFGCMOEA and variants A–J. Positive rank-biserial values favor MFGCMOEA.

Variant	IGD			HV		
	MR	$r_{\mathrm{rb}}$	n	MR	$r_{\mathrm{rb}}$	n
MFGCMOEA	1.111	–	–	**1.138**	–	–
A	6.044	0.998	45	6.280	0.998	45
B	8.773	1.000	44	8.157	1.000	44
C	8.822	0.992	43	8.481	0.989	43
D	6.684	0.992	44	6.702	0.980	44
E	6.537	1.000	45	6.825	1.000	45
F	6.198	1.000	45	6.633	1.000	45
G	6.705	1.000	34	6.713	0.997	34
H	4.455	1.000	36	4.521	1.000	36
I	4.508	1.000	36	4.543	1.000	36
J	6.164	1.000	36	6.006	1.000	36

**Table 13:** Across-problem median Spearman correlations between resource evidence and subsequent metric improvement.

Evidence	ΔIGD	ΔHV	Valid Problems
Proposed auxiliary-recruitment evidence	−0.261	−0.209	45
Feasible-offspring evidence	−0.143	−0.182	45
Main-population recruitment evidence	−0.242	−0.240	45

**Table 14:** Suite-level mechanism diagnostics of MFGCMOEA.

Diagnostic Metric	CF	DASCMOP	LIRCMOP	MW
Rank correlation	0.998	0.991	0.998	0.998
Rank MAD	0.022	0.034	0.019	0.019
Selected-set overlap	0.842	0.861	0.885	0.708
Effective neighbors	337.3	331.7	337.7	329.0
Developmental buffer	0.868	0.911	0.882	0.820
Selected infeasible rate	0.167	0.917	0.786	0.788
Conditional feasible-offspring rate	0.668	0.127	0.477	0.239

## Data Availability

The data presented in this study are available on request from the corresponding author.

## Abbreviations

**Abbreviation**	**Definition**
CMOP	Constrained multiobjective optimization problem
CMOEA	Constrained multiobjective evolutionary algorithm
MFGCMOEA	Multi-Morphogen Field-Guided Constrained Multiobjective Evolutionary Algorithm
PF	Pareto front
PS	Pareto set
CV	Constraint violation
DE	Differential evolution
FE	Function evaluation
IGD	Inverted generational distance
HV	Hypervolume
FSR	Feasible solution ratio
